# Lipidomic changes in persister cancer cells drive enhanced ferroptosis sensitivity

**DOI:** 10.70401/fos.2025.0003

**Published:** 2025-11-10

**Authors:** Eduard Reznik, Fereshteh Zandkarimi, Joleen M. Csuka, Qiulin Zhu, Jenny Jin, Taruna Vani Neelakantan, Jiewen Zheng, Vasiliki Polychronidou, Mark Fongheiser, Ashley Brown, Baiyu Qiu, Megan Rodriguez, Lela DeVine, Prem S Subramaniam, Wei Gu, Andrea Califano, Brent R. Stockwell

**Affiliations:** 1 Department of Biological Sciences, Columbia University, New York, NY 10027, USA; 2 Department of Chemistry, Columbia University, New York, NY 10027, USA; 3 Department of Systems Biology, Columbia University Medical Center, New York, NY 10032, USA; 4 Department of Pathology and Cell Biology and Herbert Irving Comprehensive Cancer Center, Columbia University Irving Medical Center, New York, NY 10032, USA; 5 Mass Spectrometry Core Facility, Columbia University, New York, NY 10027, USA; 6 Irving Institute for Cancer Dynamics, Columbia University, New York, NY 10027, USA; 7 Herbert Irving Comprehensive Cancer Center, Columbia University, New York, NY 10032, USA; 8 Department of Biochemistry and Molecular Biophysics, Vagelos College of Physicians and Surgeons, Columbia University, New York, NY 10032, USA; 9 Department of Biomedical Informatics, Vagelos College of Physicians and Surgeons, Columbia University, New York, NY 10032, USA; 10 Department of Medicine, Vagelos College of Physicians and Surgeons, Columbia University, New York, NY 10032, USA; 11 Chan Zuckerberg Biohub New York, New York, NY 10013, USA; 12 Data Science Institute, Columbia University, New York, NY 10027, USA; 13 Columbia University Digestive and Liver Disease Research Center, Vagelos College of Physicians and Surgeons, Columbia University Irving Medical Center, New York, NY 10032, USA; 14 Institute for Cancer Genetics, and Department of Pathology and Cell Biology, Herbert Irving Comprehensive Cancer Center, Vagelos College of Physicians & Surgeons, Columbia University, New York, NY 10032, USA

**Keywords:** Polyunsaturated fatty acid, diPUFA, cancer, persisters, mitochondria, ferroptosis, lipids

## Abstract

**Aims:**

Unique in the broader category of drug-resistant cells, persister cancer cells (PSs) acquire their tolerance to compounds through reversible, chromatin-mediated changes, allowing them to ‘persist’ in the face of cancer therapeutic agents. PSs are implicated in minimal residual disease from which cancer relapse occurs, and given their established sensitivity to ferroptosis, PSs present a critical point through which identification and targeting of drug-resistant cancers may be possible. Ferroptosis sensitivity in drug-resistant cancers may be caused by the attainment of the persister state, or it may merely be correlative with this state and due instead to extended inhibition of oncogenic signaling or the induction of chemotherapy stress. Nonetheless, ferroptosis sensitivity has emerged as a common phenotype across multiple PS and drug-resistant cancer cell types. Identifying biomarkers for and drivers of ferroptosis sensitivity in drug-resistant and PS cells is therefore a high priority.

**Methods:**

We derived PS cells from the lung carcinoma cell line PC9 (PS^PC9^), performed transcriptomic analysis, and subsequently lipidomics on the PC9/PS^PC9^ system. Additionally, we reverted PS^PC9^ cells to the ferroptosis-resistant parental state (PC9^PS –> PC9^) and assessed the resulting lipid changes. We generated two additional PS-like cell models: PS-like prostate carcinoma (PS^LNCaP^) from LNCaP cells and PS-like fibrosarcoma (PS^HT1080^) from HT1080 cells, with lipidomics analysis. Finally, we performed a mitochondrial elimination assay and assessed its effect on ferroptosis sensitivity.

**Results:**

We observed enrichment of lipid and sugar metabolism gene expression in PS^PC9^; lipidomics revealed enrichment within PS^PC9^ for ferroptosis-driving diPUFA phospholipids (diPUFA-PL), as well as polyunsaturated free fatty acids (PUFA FFAs). Upon PS^PC9^ reversion to the ferroptosis-resistant parental state (PC9^PS –> PC9^), this lipid signature reverted. The LNCaP and HT1080 PS-like models individually showed features consistent with PS, including an increased labile-iron pool, reversibility, and enhanced ferroptosis sensitivity, and had lipid features consistent with those in PS^PC9^. Finally, mitochondrial elimination partially abrogated ferroptosis sensitivity and altered the PS lipid profile.

**Conclusion:**

In summary, lipidomic changes dependent on the presence of mitochondria are key to the ferroptosis sensitivity of drug-tolerant persister cancer cells.

## 1. Introduction

PS cells were initially identified as a slowly-cycling drug-tolerant state achieved by a subpopulation of cells across immortalized cancer lines under pressure of a chemotherapeutic agent^[1]^. Although persister cancer cells (PSs) arise from numerous cancer tissues of origin under treatment with different chemotherapeutic agents, several conserved features of PSs have been identified, both across different tissues and within specific cell lineages^[2]^. These include non-mutational resistance to chemotherapeutics in which a subpopulation survives compound treatment and can be maintained over time; slowing or complete quiescence of the cell cycle; reversibility of the persister state; and re-acquisition of chemotherapeutic sensitivity for the majority of cells regrown from a PS population. Hangauer *et al*.^[3]^ demonstrated sensitivity of PSs to ferroptosis, a lipid-peroxidation-driven form of non-apoptotic cell death^[4,5]^, as a key feature across a variety of cancers derived from multiple tissue origins, including breast, ovary, skin, and lung.

Recently, evidence highlighting PSs as key drivers of minimal residual disease and eventual recurrence has grown^[6–11]^. The ability to identify and target this small but crucial subpopulation of cells holds potential for preventing cancer relapse. However, identification and targeting of PSs remains challenging, as there are features of both PS formation, induction, and maintenance in culture that are dynamic and potentially cell-line specific^[12]^.

Although the PS state has been identified and explored across multiple tissue origins in immortalized cancer cells^[13–15]^, the bulk of PS research has been conducted in PC9 cells^[16]^, in which it was demonstrated that a cascade initiated by mini-mitochondrial-outer-membrane permeabilization (mini-MOMP) and culminating in upregulation of activating transcription factor 4 (ATF4) drives PS^PC9^ formation^[17]^. ATF4 is a known modulator of ferroptosis sensitivity^[18,19]^, and although the ferroptosis sensitivity of PSs and the role of ATF4 in generating PS^PC9^ has been established, little is known about how PS ferroptosis sensitivity arises initially, how it is maintained during the persister state, and how it is lost when PS^PC9^ revert back to parental PC9 state (i.e., PC9^PS –> PC9^).

Recent work from our group, Qui *et al*.^[20]^, revealed a critical role for diPUFA phospholipids (diPUFA-PLs), a little-studied class of phospholipids with two polyunsaturated fatty acyl tails, in cancer cell ferroptosis sensitivity. Specifically, we reported that diPUFA-PLs interact with complex I of the mitochondrial electron transport chain (ETC) and induce mitochondrial reactive oxygen species (ROS) production that drives ferroptosis. Schwab *et al*.^[21]^ also demonstrated key mechanistic aspects of lipid profile changes in epithelial-mesenchymal transition (EMT)/plasticity-associated ferroptosis-sensitive cancer cells, such as Zeb1-modulated expression of proteins critical for monounsaturated fatty acid (MUFA) biosynthesis and polyunsaturated fatty acid (PUFA) production and incorporation into phospholipids. However, specifically in the context of PS, these lipid and mitochondrial aspects have been less explored.

Initially utilizing PC9/PS^PC9^, we identified sugar and lipid metabolism pathways as differentially expressed between PS^PC9^ and PC9 through gene set enrichment analysis (GSEA). Using liquid chromatography-mass spectrometry (LC-MS), we then found that PS^PC9^ have a ferroptosis-sensitive lipid profile enriched for diPUFA-PLs and PUFA free fatty acids (FFAs), and that this lipid profile reversed to a ferroptosis-resistant lipid composition upon PS^PC9^ reversion to PC9^PS –> PC9^ after one month. Additionally, we developed and characterized two PS-like systems, LNCaP/PS^LNCaP^ and HT1080/PS^HT1080^, that displayed multiple features consistent with PS phenotype, and measured lipidomic profile changes within each system (i.e., PS vs parental cells) and across the cell models. We also discovered that mitochondrial elimination in PS^HT1080^ partially abrogates ferroptosis sensitivity and results in changes to the PS^HT1080^ lipid architecture, revealing a key role for mitochondria in establishing the lipidomic profile of PS cells.

## 2. Methods

### 2.1 Cell models

#### 2.1.1 Cell culture and persister derivation

##### 2.1.1.1 PC9s and PS^PC9^

PC9 cells (Millapore Sigma, #90071810-1VL) from European Collection of Authenticated Cell Cultures (ECACC) were cultured in RPMI-1640 medium (ATCC, #30-2001) with 10% fetal bovine serum (FBS) (Thermo Fisher Scientific, #16140071) and 1% Pen/Strep (Thermo Fisher Scientific, #15140163) in 5% CO_2_ at 37 °C. To derive PS^PC9^, PC9 cells were trypsinized (Invitrogen, #25200-114), counted using VI-CELL analyzer (Beckman Coulter), and replated in 10 cm tissue culture treated dishes (Corning Falcon, #353003) with 15 μM erlotinib (Selleck Chemicals, #S7786) in the same media described above for a minimum of 3 days. Usual PS^PC9^ cell culture length was six days in continuous presence of erlotinib (unless stated otherwise in experiments below), after which cells were removed, counted, and used for experimental set up. Media containing the drug were fully changed every three days regardless of culture length.

##### 2.1.1.2 LNCaPs and PS^LNCaP^

LNCaP cells were cultured in RPMI-1640 medium with 10% FBS and 1% Pen/Strep in 5% CO_2_ at 37 °C. To derive PS^LNCaP^, LNCaP cells were trypsinized, counted using VI-CELL analyzer, and replated in 10 cm tissue culture treated dishes with 15 μM enzalutamide (Selleck Chemicals, #S1250) in the same medium described above for a minimum of three days. The usual PS^LNCaP^ cell culture length was six days in the continuous presence of enzalutamide (unless stated otherwise), after which cells were removed and counted. Medium with compound was changed every three days regardless of culture length.

##### 2.1.1.3 HT1080 YFP-Parkin and PS^HT1080^

HT1080 cells expressing YFP-Parkin were cultured in Eagle’s Minimum Essential Media (Quality Biological, #12-018-131) with 10% FBS and 1% Pen/Strep in 5% CO_2_ at 37 °C. To derive PS^HT1080^, HT1080 cells were trypsinized, counted using VI-CELL analyzer, and replated in 10 cm tissue culture treated dishes with 100 nM doxorubicin (ApexBio, #A3966) in the same medium described above. The typical PS^HT1080^ culture length was six days in the continuous presence of doxorubicin (unless stated otherwise). Medium with compound was changed every three days regardless of culture length. Unless specified otherwise, all HT1080 cells and PS^HT1080^ cells were cultured in the presence of 400 μg/mL Zeocin (Thermo Fisher Scientific, #R25005) to maintain selective pressure for Parkin expression.

#### 2.1.2 Cell culture

Mycoplasma tests (InvivoGen, #rep-mys-50) were routinely performed to ensure cell culture quality and all parental cell lines had no more than 20 passages. Unless specified otherwise, all 10 cm dishes used to seed and maintain PSs were initially seeded at 1 × 10^6^ cells per dish, with immediate addition of relevant compounds. All 10 cm PS cultures were maintained as separate biological replicates, derived from varying parental passage numbers, and kept in separate incubators. To generate sufficient number of PSs for any experiment, the required quantity of 10 cm dishes was generated and separately maintained. Separate cultures were pooled, counted, and plated into appropriate format (e.g., 24-well, 96-well plates) if necessary.

### 2.2 Experimental Details

#### 2.2.1 Fluorescence activated cell sorting (FACS) sorting

PC9 and PS^PC9^ were derived and maintained as described above. After six days, PS^PC9^ cells and a cohort of PC9 cells were trypsinized, washed with HBSS (Thermo Fisher Scientific, #14025092), and counted using VI-CELL. For each condition, 2 × 10^6^ cells were blocked with 10% goat serum (Thermo Fisher Scientific, #50062Z) for 30–60 minutes, stained with PE conjugated CD24 (BioLegend, #311106), APC conjugated CD133 (BioLegend, #372806), and sytox blue (Thermo Fisher Scientific, #S34857) as per vendor guidelines, then incubated in 5% CO_2_ at 37 °C in the dark. Cells were then re-suspended in HBSS. FACS was performed on a BD Influx cell sorter using BD-FACS™ (version 7.5.1.3.16) for data acquisition. FlowJo software (version 10.9.0) was used for analysis. Gating was conducted on 1 × 10^5^ events per condition (persister vs parental) first with FSC vs SSC, followed by BV421 to differentiate live from dead cells prior to PE-CD24 vs APC-CD133 analysis.

#### 2.2.2 Lipid ROS detection

PS^LNCaP^ and PS^HT1080^ were derived and maintained as described above. The same approach was taken for both cell line types: on approximately day 6 (between 5–8 days) of PS culture, both PS and a parental cell cohorts were trypsinized, counted, and equal numbers were then replated in 10 cm dishes. Cells were then treated with DMSO (Sigma-Aldrich #276855), Ras Selective Lethal 3 (RSL3), and Ferrostatin-1 (Sigma-Aldrich, #SML0583) at concentrations, combinations, and time points indicated in 5% CO_2_ at 37 °C. Cells were then collected, centrifuged at 300 g for 5 min, washed with HBSS, and incubated with HBSS containing 2.5 µM C11-BODIPY^581/591^ (Invitrogen, #D-3861) at 5% CO_2_, 37 °C for 30 min in the dark. After incubation, cells were centrifuged again, washed with HBSS, and resuspended in fresh HBSS for flow cytometry analysis using a Beckman CytoFLEX System B4-R0-V0 flow cytometer. Samples were detected with excitation at 488 nm and emission in FITC channel with 525/40 nm bandpass filter for oxidized C11-BODIPY. At least 10,000 events after gating were collected for each sample. Flow cytometry data were processed using FlowJo (version 10.9.0). Cells were gated for live cells in SSC-H vs FSC-H plot.

#### 2.2.3 Cell viability assays

##### 2.2.3.1 CellTiter-Glo luminescence assay

PSs for all cell types were derived and maintained as described above (under cell culture and persister derivation) or under specific experimental conditions as described below (e.g., PS reversion, siRNA KD, etc.). 48 h prior to CellTiter-Glo (CTG, Promega, G7573) addition, PS and a matching parental line were trypsinized, washed with HBSS, counted, and replated in either 24-well (PerkinElmer, #6005168) or 96-well (Thermo Fisher Scientific, #136101) plates at 50,000 or 10,000 cells per well, respectively. Relevant compounds and controls were added at concentrations indicated, with final volumes of 1 mL/well and 200 μL/well for 24-well and 96-well plates, respectively. After 48 h of incubation in 5% CO_2_ at 37 °C, CTG was added directly to all wells at 175 μL/well or 35 μL/well for 24 and 96-well plates, respectively. Plates were placed on an orbital shaker at low speed for approximately 15 minutes, and luminescence was measured using a Victor X5 plate reader (PerkinElmer). All cell viability data were normalized to the DMSO vehicle condition. DMSO concentration did not exceed 0.3% in any experiment and always matched the highest percentage of DMSO for any drug treatment. GraphPad Prism (version 10) was used for visualization and statistical analysis.

##### 2.2.3.2 Vi-cell trypan exclusion dye cell counter

At the indicated time points using VI-CELL count, cells were trypsinized, spun down at 300 *g* in a Sorval ST40 centrifuge (Thermo Fisher Scientific) for five minutes, resuspended in HBSS, and counted using VI-CELL. GraphPad Prism (version 10) was used for visualization and statistical analysis.

##### 2.2.3.3 Giemsa staining

Parental lines of each cell type were prepared to be stained on the same day as the corresponding PS cohort. 3.8 g Giemsa powder (Sigma-Aldrich, #G9641-5G) was added to 250 mL methanol (Honeywell, #34966), heated to 60 °C, and 250 mL glycerol (Sigma-Aldrich, #G5516) was added slowly. The solution was cooled and filtered, and aliquots were dissolved in phosphate buffered saline (PBS) to a final concentration of approximately10% Giemsa. At the indicated time points, cells were washed with approximately 3 mL methanol for five minutes, the methanol was removed and cells were then incubated with 10% Giemsa for approximately 30 minutes on an orbital rotator at low speed. When ready, Giemsa reagent was removed, and 10 cells were photographed against a white background using a smartphone camera or imaged on a ChemiDoc MP (BioRad) using the 590/110 nm Coomassie blue setting.

#### 2.2.4 Labile iron pool

At six days, 200,000 cells from each model, PS^HT1080^ and HT1080, were collected, washed twice with PBS, resuspended in 500 nM of calcein-AM (AAT Bioquest, #22002) and incubated at 37 °C in the dark for 20 minutes. Cells were then centrifuged at 300 g for 5 minutes and washed with PBS. Cells were resuspended in PBS followed by flow cytometry analysis using a Beckman CytoFLEX System B4-R0-V0 flow cytometer. Cells were gated for live cells in SSC-A vs. FSC-A, and for single cells in FSC-H vs FSC-A, and then analyzed on the FITC channel. 10,000 events were recorded. Data analysis was performed using FlowJo (version 10.9.0)

#### 2.2.5 PI staining

At six days, three million cells from each model (PS^HT1080^ and HT1080) were collected and resuspended in 1 mL PBS at retention times (RT), then transferred into 4 mL ethanol at −20 °C for 15 minutes. Cells were centrifuged at 300 g for 5 minutes and then resuspended in 5 mL PBS. After 15 minutes at 4 °C, cells were centrifuged again at 300 g for 5 minutes and then resuspended in 0.5 mL FxCycle PI/RNAse Staining Solution (Thermo Fisher, #F10797). Samples were gently vortexed and incubated for 20 min at RT in the dark. Samples were analyzed using the Beckman CytoFLEX System B4-R0-V0 flow cytometer. Cells were gated for live cells in SSC-A vs FSC-A, and for single cells in FSC-H vs FSC-A, then analyzed on the PE channel. 10,000 events were recorded. Data analysis was performed using FlowJo (version 10.9.0)

#### 2.2.6 Exogenous diPUFA-PL addition

At six days, parental HT1080 cells or PS^HT1080^ cells were seeded in the 96-well plate at a density of 10,000 cells/well. Before treatment, a lipid stock solution in chloroform was dried under nitrogen flow, then re-dissolved in vehicle containing 20% ethanol and 16% 2-hydroxypropyl-β-cyclodextrin (Cayman Chemical, #16169) in PBS to prepare a 30 mM solution, and sonicated until the solution was clear. Phospholipids were further diluted in cell culture medium to reach desired concentrations and then added to cell plates. The final vehicle concentration in cells was less than 0.02%. RSL3 was added at the indicated concentrations. After 24 h of phospholipid and RSL3 treatment, CTG was added to the plate, and cell viability was measured.

#### 2.2.7 Western blot

Once collected via trypsinization, cells were centrifuged, resuspended in HBSS and counted. Equal number of cells per condition were aliquoted, washed again with HBSS, pelletized at 300 g for 5 min, and lysed in 80 μL RIPA buffer (Thermo Fisher Scientific, #89901) containing protease inhibitor cocktail (Sigma-Aldrich, #11697498001), then incubated on ice for 20 min. The lysate was centrifuged at 17,000 g for 15 min at 4 °C. The supernatant was transferred to a new tube and a BSA assay was performed, followed by normalization of all conditions for both protein concentration and volume. 4x Laemmli buffer containing 125 µM DTT (Cell Signaling Technology, #7722S) was added to all samples, which were incubated at 100 °C for 10 min. An equal amount of protein in the range of 45–80 µg was loaded in each lane of the NuPAGE 4–12% Bis-tris gel (Thermo Fisher Scientific, #WG1401) and transferred onto nitrocellulose membranes (Thermo Fisher Scientific, #IB23002) using iBlot2 (Invitrogen) electrophoretic semi-dry western blot transfer system. Membranes were blocked with PBS blocking buffer (LI-COR Biosciences, #927-70001) for 1 h at room temperature and incubated with primary antibodies diluted in 1:1 PBS-T (PBS with 0.1% Tween20): blocking buffer at concentration of 1:250 for protein of interest or 1:5000 for control, overnight at 4 °C. Primary antibodies used were ATF4 rabbit mAb (Cell Signaling, #11815), GPX4 rabbit mAb (Abcam, #125066), GCH1 rabbit (Bethyl Laboratories, # A305-296A-M, product has since been discontinued), cytochrome c rabbit mAb (Cell Signaling, #11940S) and *β*-actin mouse mAb (Cell Signaling, #3700). Membranes were washed three times in PBS-T and then incubated with secondary antibodies IRDye 800CW goat anti-rabbit IgG (LI-COR Biosciences, #926-32211) and IRDye 680RD donkey anti-mouse IgG (LI-COR Biosciences, #926-68072) diluted at 1:5,000 and 1:20,000, respectively, in 1:1 PBS-T: blocking buffer for 1 h at room temperature. Membranes were washed five times in PBS-T and imaged using the LI-COR Odyssey infrared imaging system. Images were collected and optimized using the Image Studio software. Fiji 2 software (version 2.9.0/1.53t) was used for quantification.

#### 2.2.8 Bulk RNAseq

At six days, after washing with HBSS, total RNA was extracted from cells using the AllPrep DNA/RNA Isolation Kit (Qiagen, #80204). Total RNA was quantified using a NanoDrop™ Lite Spectrophotometer (Thermo Fisher Scientific, #ND-LITE-PR). Following RNA quality verification via bioanalyzer, poly-A pull-down was used to enrich mRNAs from total RNA, and library construction was performed using Illumina TruSeq chemistry. Libraries were then sequenced on the Element AVITI at the Columbia Genome Center with multiplexed samples in each lane, yielding approximately 40 million paired-end 75bp reads for each sample. The tool bases2fastq (version 1.1.0) was used for converting BCL to fastq format, coupled with adaptor trimming. FASTP (version 0.23.3)^[22]^ was used for read quality control using default settings and Kallisto (version 0.46.1)^[23]^ was used to perform pseudoalignment with bootstrapping to an index created from transcriptomes (Ensembl v96, Human:GRCh38.p13).

#### 2.2.9 Combining gene expression data, batch effect identification and correction

Three sets of randomly selected parental and persister fastq files from the Green lab data were downloaded from Gene Expression Omnibus (GEO) (accession GSE189625, SRRs 69,71,73,75,77, 115, 117, 119, 127, 129, 131) and processed using the same software pipeline described above. Sleuth (version 0.30.0)^[24]^ was used for differential gene expression analysis. Briefly, a likelihood ratio test and a Wald test with the b statistic modified to be mathematically equivalent to log_2_ of the fold change were performed between two models (reduced and full) in transcript and gene mode. Although Sleuth 0.30.0 offers an option to aggregate on *P*-value from transcript differential expression, we assessed and corrected for batch effect (here means data from different labs are treated as two separate batches) as described in previous version of the Sleuth online tutorial, while retaining a measure of effect size via fold change (specifically, the Wald statistic) for incorporation into GSEA ranking along with significance. A batch-naïve full vs. reduced model was first fit, and PCA analysis of the top 3 principal components revealed a batch effect in PC1 vs PC2. A second model specifically taking batch as a covariate into account for full model vs reduced model then fit, and the differences between naïve vs batch corrected analyses were quantified as described in the tutorial. All subsequent analysis was performed on batch corrected data.

#### 2.2.10 GSEA of combined batch-corrected data

The full list of genes from the differential gene expression analysis was then ranked by incorporating *P*-value and fold change (b statistic of Wald test, modified to be mathematically equivalent to fold change) using the formula: *−log_10_(p.value) × log_2_(FC)*. GSEA was performed on the ranked gene list utilizing the Kyoto Encyclopedia of Genes and Genomes (KEGG) database and Fast Gene Set Enrichment Analysis (FGSEA) method^[25,26]^ in clusterProfiler (version 4.6.2)^[27,28]^.

#### 2.2.11 Untargeted lipidomics

##### 2.2.11.1 Lipidomic sample preparation

PSs for all cell line types were derived and maintained as described above (under cell culture and persister derivation) and collected on day 6. The 15- and 34-day PC9^PS –> PC9^ cohorts were timed to be ready to collect at the same time as 6-day PS, along with a cohort of parental cells. All cells were maintained in 10 cm dishes, and 15- and 34-day PC9^PS –> PC9^ reverters were passaged as necessary to avoid over confluence while reaching 15 and 34 days post erlotinib removal, respectively. Cells with and without mitochondria were generated as described in the mitochondrial elimination section below. For all samples, three million cells per replicate were used. For lipid extraction, the procedure was adapted from previously described methods^[29]^. Briefly, cells were scraped in 3 mL of ice-cold PBS containing butylated hydroxyl toluene (BHT, 0.001% w/v in ethanol) and transferred to cold 5 mL tubes. After vortexing, a 300 μL aliquot was taken for protein analysis and the remaining volume (approximately 1.7 mL) was centrifuged at 350 g for 5 min at 4 °C. After centrifugation, the supernatant was aspirated and the pellet stored at −80 °C. The next day, samples were mixed with 1 μL of SPLASH lipidomics internal standard mix (Avanti Polar Lipids, Inc.) with a microtip ultrasonicator. After homogenization, samples were transferred to glass vials containing 850 μL of cold methyl-tert-butyl ether and vortexed for 30 s. A 2 h incubation (on ice) was performed to improve lipid extraction efficiency. Thereafter, 200 μL of ice-cold water was added, and the samples were incubated on ice for 20 min. After centrifugation (3,000 r.p.m. for 20 min at 4 °C), the lipid-containing upper phase was collected and dried under a gentle stream of nitrogen gas. A mixture of 2-propanol/acetonitrile/water (4:3:1, v/v/v and 0.01% butylated hydroxytoluene) was used to reconstitute the dried samples before LC-MS analysis. A quality control sample was prepared by combining 40 μL of each sample to assess the reproducibility of features throughout the chromatography runs.

##### 2.2.11.2 LC-MS conditions on SYNAPT G2

Samples were analyzed on a SYNAPT G2 LC-MS instrument. Lipids were separated using an Acquity UPLC CSH column (2.1 × 100 mm, 1.7 μm) over a 20-min gradient elution on a Waters Acquity UPLC I-Class system. Mobile phases A [acetonitrile/water (60:40, v/v)] and B [2-propanol/acetonitrile/water (85:10:5, v/v/v)] contained 0.1% acetic acid and 10 mM ammonium acetate. Following injections, the gradient was held at 40% mobile phase B for 2 min. At 2.1 min, it reached to 50% B, then increased to 70% B over 12 min; at 12.1 min, it was held at 70% B and at 18 min increased to 90% B. The eluent composition returned to the initial condition in 1 min, and the column was re-equilibrated for an additional 1 min before the next injection. The column oven temperature was set at 55 °C and the flow rate was 400 µL·min^−1^. Flow-through needle mode was used to allow for injections of 6 µL. Quality control samples were injected between the samples and at the beginning and end of the run to monitor the performance and the stability of the instrument. The SYNAPT G2-Si Q-ToF mass spectrometer was operated in both positive and negative electrospray ionization modes. For positive mode, a capillary and sampling cone voltages of 2 kV and 32 V were used. Source and desolvation temperatures were kept at 120 and 500 °C, respectively. Nitrogen was used as the desolvation gas with a flow rate of 800 L·h^−1^. For negative mode, the capillary voltage of 1.5 kV and the cone voltage of 30 V were used. The source temperature was 120 °C and the desolvation gas flow was set to 800 L·h^−1^. Depending on ionization mode, the protonated molecular ion of leucine encephalin ([M + H]^+^, mass to charge ratio (*m*/*z*): 556.2771) or the deprotonated molecular ion ([M − H]^−^, *m*/*z*: 554.2615) was used as a lock mass for mass accuracy and reproducibility. Data were collected in duplicates in data-independent (MS^E^) mode over the mass range *m*/*z*: 50 to 1,200 Da. The quality control samples were also acquired in enhanced data-independent ion mobility (HDMSE) in both positive and negative modes to aid structural assignment of lipid species. Electrospray ionization source settings for ion mobility were the same as described above. The traveling wave velocity was 650 m·s^−1^ and the wave height was 40 V. The helium gas flow in the helium cell region of the ion-mobility spectrometry cell was set to 180 ml·min^−1^. Nitrogen, used as the drift gas, was held at a flow rate of 90 ml·min^−1^ in the ion-mobility spectrometry cell. The low collision energy was 4 eV and the high collision energy was ramped from 25 to 60 eV in the transfer region of the T-Wave device to induce the fragmentation of mobility-separated precursor ions.

Some experiments were performed on a Synapt-XS mass spectrometer coupled to an Acquity UPLC system (Waters Corp). LC-MS parameters were adapted from the above methods^[30]^. Chromatographic separation was performed over a 20-minute gradient elution profile on an Acquity Premier Peptide CSH C18 Column (1.7 μm, 2.1 × 100 mm) (Waters Corp) at 65 °C. Both positive and negative ionization modes were used to analyze all samples. The binary mobile phase consisted of solvent A (60:40 v/v acetonitrile: water) and solvent B (85:10:5 v/v/v/ isopropanol: acetonitrile: water), each containing 10 mM ammonium acetate and 0.1% acetic acid. The solvent gradient began with 40% solvent B. In 2 minutes, there was a linear gradient to 50% solvent B. Solvent B then ramped up to 99% over the next 18 minutes. The next 2 minutes were for column equilibration back to 40% solvent B. Samples were also run using high-definition data independent mode with ion mobility (HDMS^E^). Additional parameters include a flow rate of 400 µL/min, injection volume of 4 µL for positive mode and 8 µL for negative mode, mass range of m/z 50 to 1,200 Da, scan rate of 0.1 sec/scan, 2.0 kV capillary voltage, and 40.0 kV sampling cone voltage. Desolvation temperature was set to 450 °C and desolvation gas flow was 800 L/h.

##### 2.2.11.3 Untargeted lipidomic data preprocessing and analysis

The structural elucidation and validation of significant features were obtained by searching monoisotopic masses against the available online databases, such as METLIN, Lipid MAPS and HMDB, with a mass tolerance of 5 ppm. Fragment ion information obtained by tandem MS (UPLC-HDMS^E^) was used for the further structural elucidation of significantly changed lipid species. HDMS^E^ data were processed using MSE data viewer (version 1.3, Waters Corp). Raw data files were processed in Progenesis QI software. Multivariate and univariate statistical analyses were performed using MetaboAnalyst (version 5.0)^[31]^ and also a local implementation in an R environment^[32]^. Group differences were calculated using one-way analysis of variance (ANOVA). P values were corrected for multiple hypothesis testing and a false discovery rate (FDR) of 0.05 or less was considered significant.

#### 2.2.12 Targeted lipidomics

Standards were acquired from Avanti Polar Lipids (PC(18:2_18:2), #850385C; PC(22:6_22:6), #850400C; PC(20:4_20:4), #850397C) and suspended in the same 4:3:1 solvent mixture used for the samples described in untargeted lipidomics section. Standards were run for the prostate carcinoma and fibrosarcoma samples under identical LC-MS analysis conditions described previously. Quantification of lipid abundance was performed via TargetLynx within MassLynx (version 4.2) and statistical analysis and visualization were performed in GraphPad Prism (version 10).

#### 2.2.13 siRNA KD

PS^PC9^ cells were derived and maintained as described above (under cell culture and persister derivation). Cells were transfected by reverse transfection using Lipofectamine RNAiMAX Transfection Reagent (Thermo Fisher Scientific #13778075) according to the manufacturer’s protocol. Briefly, 80 pmol of siRNA ON-TARGETplus human ATF4 siRNA SMARTpool (Dharmacon, #L-005125-00-0005) or ON-TARGETplus non-targeting control siRNA, (Dharmacon, #D-001810-01-05) and 15 μL Lipofectamine RNAiMAX were mixed in 2 mL Opti-MEM reduced serum medium (Life Technologies, #31985) at room temperature for 30 min. Then, 1 × 10^6^ cells in 8 mL media were mixed with 2 mL transfection reagent and added to 10 cm dish. After 48 h, cells were collected for analysis.

#### 2.2.14 CRISPR/Cas9 ATF4 KO

Cas9 and ATF4 sgRNA expressing plasmid (ABM, #126101110595) was heat shock transformed into DH5α competent *E. coli* cells (Thermo Fisher Scientific, #18258012), followed by selection and expansion using ampicillin (IBI Scientific, #IB02040) as per manufacturer’s recommendations. Bacteria were then Mira prepped^[33]^ using Qiagen spin mini prep kit (Qiagen, #27104) reagents, and plasmid was quantified on Nanodrop spectrophotometer (Thermo Fisher Scientific). ATF4/Cas9-containing virus was generated using HEK293T cells. Briefly, HEK293T cells (ATCC, #CRL-3216) cultured in DMEM (Cytiva, #SH30243) with 10% FBS were reverse transfected using the siRNA protocol described above, but using plasmid concentrations of 5 μg ATF4, 5 μg Gag-Pol (Addgene, #12260), and 1 μg VSV-G (Addgene, #8454) for every 15 μL Lipofectamine. After 24 h incubation in 5% CO_2_ at 37 °C, the medium was changed to 100% HEK293T media and cells were incubated in 5% CO_2_ at 32 °C. Virus medium was collected every 24 h for 2 days and pooled. Lenti-X (Takara Bio, #631231) concentrator was then used as per manufacturer’s protocol to increase virus concentration by25 fold. PC9 and PS^PC9^ (derived and maintained as described above under cell culture and persister derivation) were infected with and maintained in virus containing media for 36 hours. Upon removal of viral media, cells were maintained in selection agent puromycin (Santa Cruz Biotechnology, #sc-108071A) for the duration of experiment until collection and counting for viability assay.

#### 2.2.15 Mitochondrial elimination

Mitochondrial elimination was performed as previously stated in Gaschler *et al*.^[34]^. Briefly, HT1080 cells and PS^HT1080^ cells were cultured as described above (under cell culture and persister derivation). After 5 days in culture, 12.5 μM of carbonyl cyanide m-chlorophenyl hydrazone (CCCP) (Cayman Chemical, #25458) was added to PS^HT1080^ cells, and incubated for an additional 48 h. HT1080 cells receiving CCCP were trypsinized, counted, replated in 10 cm plates, and subjected to the same CCCP treatment conditions. After 48 h, cells were trypsinized, counted, and used in experiments in CCCP-free medium.

#### 2.2.16 *In vivo* studies

All experimental procedures involving mice were reviewed and approved by the Columbia University Institutional Animal Care and Use Committee. The mice were monitored daily and euthanized when they exhibited any of the following: peri-orbital hemorrhages, epistaxis (nose bleeds), seizures, decreased level of alertness and activity, impaired motor function, and/or impaired ability to feed secondary to decreased motor function, paresis or coma, loss of more than 20% of baseline body weight, or tumor volumes surpassing 2,000 cubic millimetres.

##### 2.2.16.1 Animals, tumor xenograft model, and drug administration

Immunodeficient nude mice (NSG, Jackson Laboratories) were received at 7 weeks age and were allowed to acclimate for at least three days. Mice (*n* = 60, female) were injected subcutaneously in the right flank with 5 million PC9 non-small lung cell carcinoma cells at 8 weeks of age. Mice were monitored over 2–4 weeks, and five mice at a time were recruited into treatment arms once tumor size reached 100–300 mm^3^, maintaining similar average tumor sizes across groups. Recruited mice were weighed, and tumor sizes were measured daily prior to injection. All injections were prepared fresh on the day of administration and sterile-filtered through a 0.22 µm syringe filter. Mice were injected intraperitoneally (IP) with VP-224 (15 mg/kg), vehicle (5% DMSO, 80% PBS, 10% ethanol, 5% Kolliphor EL) or with erlotinib (100 mg/kg) via oral gavage daily for 10 days for the first treatment leg, followed by a 2-day treatment break. The second treatment leg included the appropriate combination of the same daily injections over 10 days. Euthanasia via CO_2_ asphyxiation and cardiac puncture was performed on mice four hours after the final injection, with plasma collected and tumor extracted and weighed. Mice exhibiting early endpoint signs (loss of > 20% body weight, tumor size > 2,000 mm^3^, decreased motor function, impaired breathing) were euthanized via CO_2_ asphyxiation and cervical dislocation.

##### 2.2.16.2 Immunohistochemistry (IHC) staining in lung cancer xenograft paraffin section

For deparaffinization and hydration, three rounds of xylene wash for 5 minutes, followed by two rounds with 100% EtOH and 1 round of 50% EtOH for 5 minutes each, with a final wash in dH_2_O was conducted. For antigen retrieval, 1 L of 0.01 M citrate buffer (PH6) was used in a pressure cooker with slides in microwave for 8 minutes on full power then 8 minutes on reduced power. The pressure cooker was then removed from the microwave, cooled for ten minutes at RT with lid on, then opened and cooled for an additional 30 minutes. Slides were washed with dH_2_O and then PBS-T three times for five minutes each. Endogenous peroxidase was quenched in 3% hydrogen peroxide in PBS-T for 10 min, then the slides were washed in dH_2_O, and PBS-T three times for five min; 10% normal horse serum for 30 min was used for blocking. Serum was removed and primary antibodies were added to the sections for an incubation period of 1 h at RT. Primary antibodies used were: CD24 at 1/100 invitrogen #MA5-11828 (tonsil for positive control) and CD133 1/50 Invitrogen #MA1-219 (pancreas for positive control). Antibodies were removed, washed in PBS-T three times for five min each, and horse anti-mouse biotinylated 1/200 (Vector) added to the section, with subsequent incubation for 30 min at RT. Slides were again then washed in PBS-T three times for five min each, followed by addition of avidin-biotin complex to the sections, and incubation for 30 minutes at RT. They were then washed again in PBS-T three times for five minutes each and DAB solution (DAKO) was added to each section. Stains were developed and immersed in dH_2_O, then counterstained with Hematoxylin, dehydrated, and cover slips mounted.

### 2.3 Statistics

Statistical analyses were performed using GraphPad Prism 10, Sleuth 0.30.0, clusterProfiler 4.6.2, and Metaboanlayst 5.0 (which software used to analyze which experiment specified in relevant methods section). For each experiment, the particular statistical analysis is detailed in the respective figure legend. Unpaired student’s T-test, one-way ANOVA, or two-way ANOVA with appropriate post-hoc tests were performed on approximately normally distributed data. All statistical testing was two-tailed.

### 2.4 Resource availability

#### 2.4.1 Materials and correspondence

Further information and requests for resources and reagents should be directed to and will be fulfilled by the Lead Contact, Brent R. Stockwell (bstockwell@columbia.edu).

## 3. Results

### 3.1 PC9/PS^PC9^ model is consistent with prior observations of PS features

We sought to confirm that our PC9/PS^PC9^ cell model was consistent with previous observations. Notably, a 14-day time point analyzed by Oren *et al*.^[35]^ elucidated that cycling and non-cycling persisters can originate from different cell lineages and have their distinct transcriptional and metabolic programs. To preclude effects from fast-cycling persisters, unless stated otherwise, we maintained PC9 cells in the presence of erlotinib for six days, after which cells were collected and features were experimentally assessed, prior to the potential for fast cycling cells to dominate the culture.

While no definitive individual marker for PS cells has been found^[12]^, numerous phenotypic characteristics have been documented regarding PC9/PS^PC9^ cells, including but not limited to predominance of CD24 and CD133 in PS^PC9^, reversibility of PS state by removal of erlotinib from media, and expansion of cell number by passaging, resensitization of the majority of reverted cells to erlotinib, and sensitivity of PS^PC9^ to ferroptosis compared to PC9 cells.

We found all of these features to be present in PC9 cells after six days in erlotinib (PS^PC9^): PS^PC9^ cells were CD24 and CD133 positive versus PC9 cells (Figure S1a). PS^PC9^ were sensitive to ferroptotic cell death induced by RSL3 that was prevented by treatment with the ferroptosis inhibitor Ferrostatin-1 (Fer-1) but not by ant-apoptotic agent Z-VAD-FMK (Z-VAD) (Figure S1b). PS^PC9^ cells were resistant to erlotinib but sensitized to ferroptosis vs PC9 (Figure S1c). To assess the reversibility of cell growth, we maintained multiple 10 cm dishes of PS^PC9^, and at day 11, erlotinib was removed from several of the PS^PC9^ plates (termed PC9^PS –> PC9^). Then, the cells were continued to maintain for 10 more days. By day 21, the difference between PS^PC9^ and PC9^PS –> PC9^ was clear: PC9^PS –> PC9^ cells without erlotinib present for 10 days proliferated substantially more than PS^PC9^ cells (Figure S1d,e) and, upon re-introduction of erlotinib for 2 days to PC9^PS –> PC9^, the cells again died, as visualized by Giemsa staining (Figure S1d). Although given the 21-day time frame, it is possible a fast-cycling persister sub-population was present within the erlotinib-maintained PS^PC9^ cell culture, the erlotinib-deficient PC9^PS –> PC9^ cells proliferated at a rate even greater than that, resulting in cell number differences by day 21. While it has previously been shown that PC9^PS –> PC9^ reacquire ferroptosis resistance by approximately 2 months^[3]^, we found that PC9^PS –> PC9^ cells displayed this ferroptosis-resistant phenotype by day 30 post erlotinib removal (Figure S1f).

Collectively, these data indicate that this PS^PC9^ cell model is: (1) reversible by erlotinib removal, (2) resistant to erlotinib, (3) sensitive to ferroptosis, (4) slowly-cycling at a rate substantially lower that of both PC9 and PC9^PS –> PC9^, (5) enriched for CD24 and CD133^[1]^, and (6) reacquires ferroptosis resistance upon reversion to PC9^PS –> PC9^. We therefore conclude that six days in erlotinib generated a PC9/PS^PC9^ cell model exhibiting key features of drug-tolerant persisters.

### 3.2 Gene set enrichment analysis of PC9/PS^PC9^ cells reveals lipid and sugar metabolism changes

**Figure 1.**
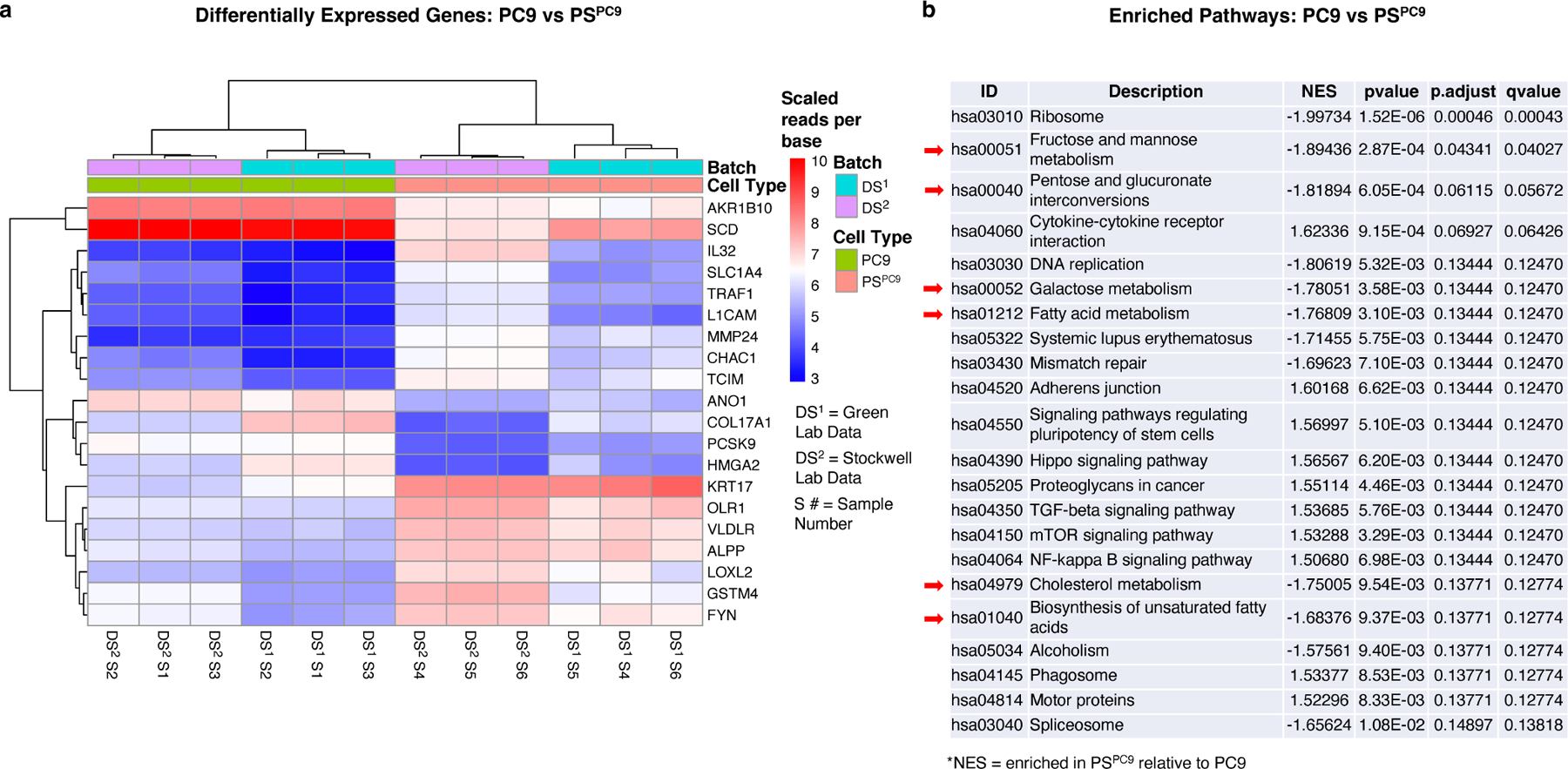
GSEA of PC9/PS^PC9^ reveals lipid and sugar metabolism as a key PS/parental differential. (a) The top 20 DGEs comparing PC9 vs PS^PC9^ with batch effect corrected in the combined gene expression data. Colored by scaled reads per base and generated using the Sleuth “plot_transcript_heatmap” function with transcripts clustered (Jensen-Shannon distance, hierarchical clustering of rows and columns). BH-corrected significance (all have corrected P < 10^−6^). n = 9 independently cultured, erlotinib-treated biological replicates of PS, pooled into three replicates; n = 3 independently-cultured, biological replicates of PC9 for Stockwell Lab samples; (b) GSEA on ranked list of around 17,000 genes from DGE analysis, red arrows highlight abundance of sugar or lipid metabolic pathways exceeding significance threshold. NES is ± in PS^PC9^, relative to PC9. BH corrected P < 0.15 displayed. GSEA: gene set enrichment analysis; DGE: differential gene expression; PS: Persister cancer cell; BH: Benjamini-Hochberg; NES: normalized enrichment score.

To identify gene expression changes associated with the differential ferroptosis sensitivity of PC9/PS^PC9^, we conducted transcriptomics analysis. PS^PC9^ cells were generated in 10 cm dishes as described above, and at day six, cells were collected and RNA extraction, and sequencing was performed. Moreover, in light of Kalkavan *et al*. reporting the role of ATF4 in the PS^PC9^ state^[17]^, we downloaded the PC9 and PS^PC9^ RNAseq fastqs deposited in the GEO database^[17]^, and after identifying and correcting for batch effects in the combined lab data sets (Figure S2a, Table S1), we performed differential gene expression (DGE) analysis, followed by GSEA on the combined data (Figure 1a,b). After batch correction, samples clustered according to their biological conditions across all significant genes regardless of lab location in which the data were generated (Figure S2b,c, Figure 1a). The top 20 DGEs includes genes related to or involved in ferroptosis and its mitigation (e.g., CHAC1, VLDLR, COL17A1, SCD)^[18,36–38]^; other ferroptosis genes were absent (e.g., SLC7A11, GPX4)^[4,39]^.

GSEA allows for the detection of subtle, pathway-wide transcriptional changes, highlighting pathway regulation shifts that may be missed by lists of genes^[40,41]^. GSEA guidelines recommend a FDR < 0.25 for exploratory analysis of a limited number of gene sets analyzed using standard parameter settings (phenotype permutation, min to max set size boundaries of approximately 15 to 500, respectively). Using KEGG^[25,42]^ and applying boundaries of 20 to 500, we observed 26 out of approximately 355 human pathways (hsa pathways) were enriched in the combined data. Additionally, we noticed that regardless of number of re-analyses, the majority of pathways consistently exhibited FDR values around 0.13 or lower, including pathways previously identified as differential between persisters and parentals^[17,35]^, in the same direction as previously observed (*e.g.*, NF-Kappa β signaling, cytokine-cytokine receptor interaction, DNA replication). We observed a preponderance of negatively enriched metabolic pathways in PS^PC9^ over PC9 (Figure 1b, red arrows), including fructose and mannose, pentose and glucoronate interconversion, galactose, biosynthesis of unsaturated fatty acids, fatty acid metabolism, and cholesterol metabolism. Collectively, these data are consistent with previous observations and suggest a differential signature in lipid and sugar metabolism between parental and persister cells. These findings prompted us to pursue lipidomics to further investigate the specific lipid and metabolite differences between the parental and persister state.

### 3.3 Untargeted lipidomics analysis of PC9/PS^PC9^ cells reveals that PS have a ferroptosis-sensitive lipid profile enriched for PUFA lipids

**Figure 2.**
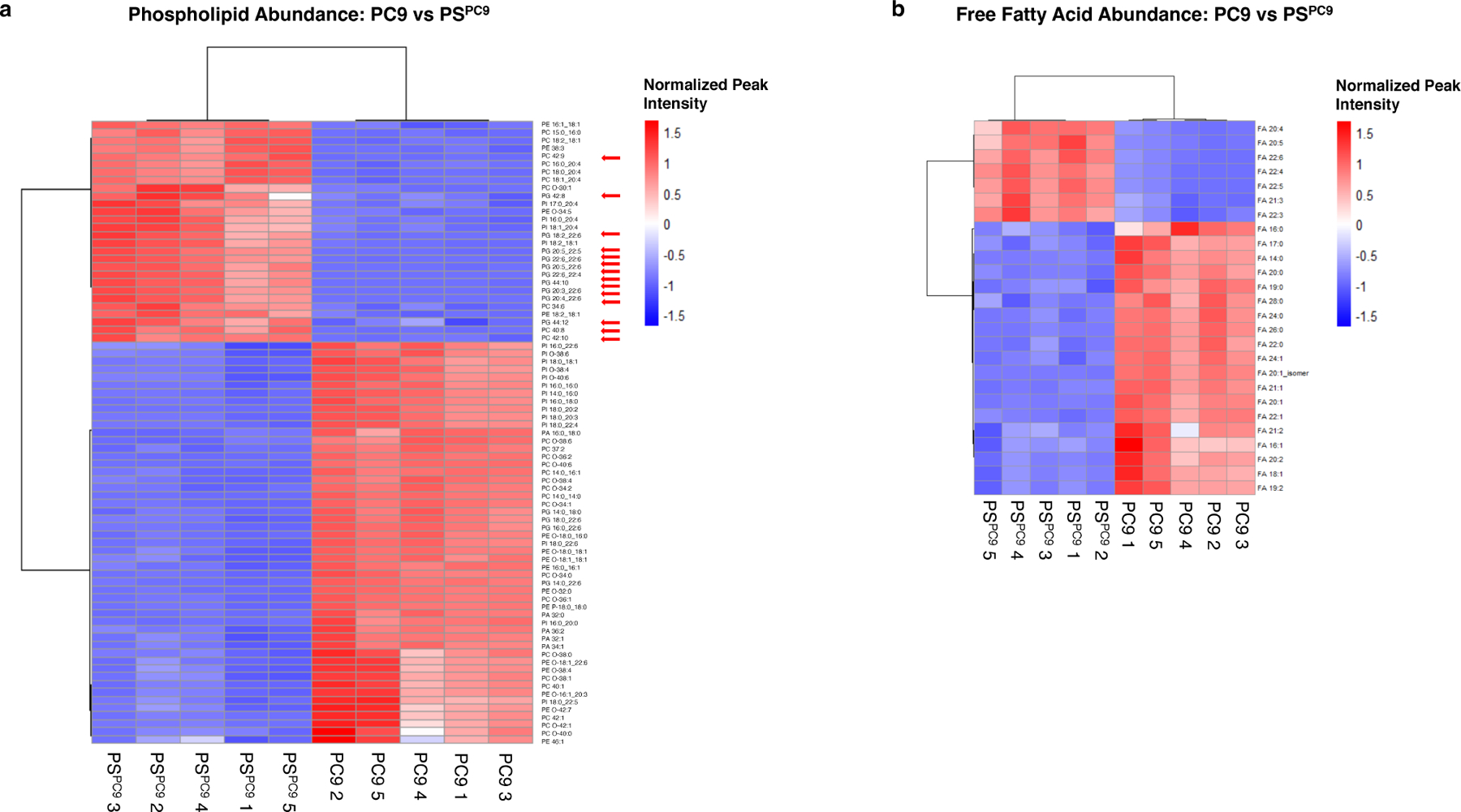
Untargeted lipidomics analysis of PC9/PS^PC9^ cells reveals that PSs have a ferroptosis-sensitive lipid profile enriched for diPUFA phospholipids. (a) Abundance of 79 PLs and (b) 26 FFAs changed significantly (FDR < .05) in PC9 vs PS^PC9^. Red arrows highlight diPUFA-PLs. 3 × 10^6^ cells per sample for all cell types were plated. Independently cultured, erlotinib-treated biological replicates of PSs cultured in sufficient quantity to pool into n = 5 replicates; n = 5 independently cultured biological replicates of PC9. One-way ANOVA of normalized peak intensity with Euclidean distance and Ward clustering on rows and columns. PSs: persister cancer cells; diPUFA: di-polyunsaturated fatty acid phospholipids; FDR: false discovery rate; FFAs: free fatty acids; ANOVA: analysis of variance; PLs: phospholipids.

Guided by the transcriptomics results, we interrogated the lipidomic profiles of PC9 versus PS^PC9^ cells using untargeted lipidomics. PS^PC9^ cells were generated in the same 10 cm format described above, and at day six, samples of PS^PC9^ and PC9 were collected, lipids extracted, and LC-MS analysis was performed. Globally, without applying any filtering or statistical significance testing, PC9 vs PS^PC9^ samples clustered according to their biological conditions and displayed starkly contrasting lipid profiles (Figure S3a). Upon applying filtering and significance testing, we observed that although PS^PC9^ cells contain an abundance of mixed-acyl phospholipids with a single polyunsaturated fatty acyl tail (monoPUFA-PLs) relative to PC9 cells, they were also enriched in a class of PLs that we recently identified as drivers of ferroptosis^[20]^: diPUFA-PLs (Figure 2a, red arrows). Conversely, parental PC9 cells were enriched in phospholipids with saturated fatty acid tails.

Additionally, we observed an abundance of polyunsaturated free fatty acids (PUFA FFA) in PS^PC9^ versus PC9 cells, consistent with higher diPUFA PL (Figure 2b). We found a depletion of ferroptosis-blocking monounsaturated and saturated FFA in PS^PC9^ cells, consistent with their increased sensitivity to ferroptosis. PS^PC9^ persister cells thus have a lipidomic profile enriched in lipids susceptible to peroxidation, a class of which has been identified as a driver of ferroptosis, whereas parental PC9 cells do not.

### 3.4 Untargeted lipidomic analysis of PC9/PS^PC9^ cells during PS reversion to parental cells reveals re-acquisition of ferroptosis-resistant lipidome

To determine whether the lipid profile differences between persister PS^PC9^ and parental PC9 cells correlated with ferroptosis sensitivity, we conducted a multi-timepoint untargeted lipidomic analysis comparing parental PC9, persister PS^PC9^, and reverted PC9^PS –> PC9^ cells. We observed one of the major phenotypic features of parental reversion, the reacquisition of ferroptosis resistance, occurs one month post erlotinib removal (Figure S1f); therefore, we selected 15 days as an intermediate point to assess lipid profile changes during this dynamic process.

PS^PC9^ cells were generated as described above, and after six days, erlotinib was removed from the medium (generating cells termed PC9^PS –> PC9^). Batches of PC9^PS –> PC9^ cells were thus free of erlotinib for either 15 or 34 days and collected simultaneously with a cohort of PC9 cells and another cohort of 6 days of erlotinib-treated PS^PC9^ cells. Thus, four experimental conditions were generated (parental PC9; PS^PC9^; 15 day post erlotinib PC9^PS –> PC9^; 34 day post erlotinib PC9^PS –> PC9^), but all collected concurrently, lipids extracted, and lipidomics was performed.

**Figure 3.**
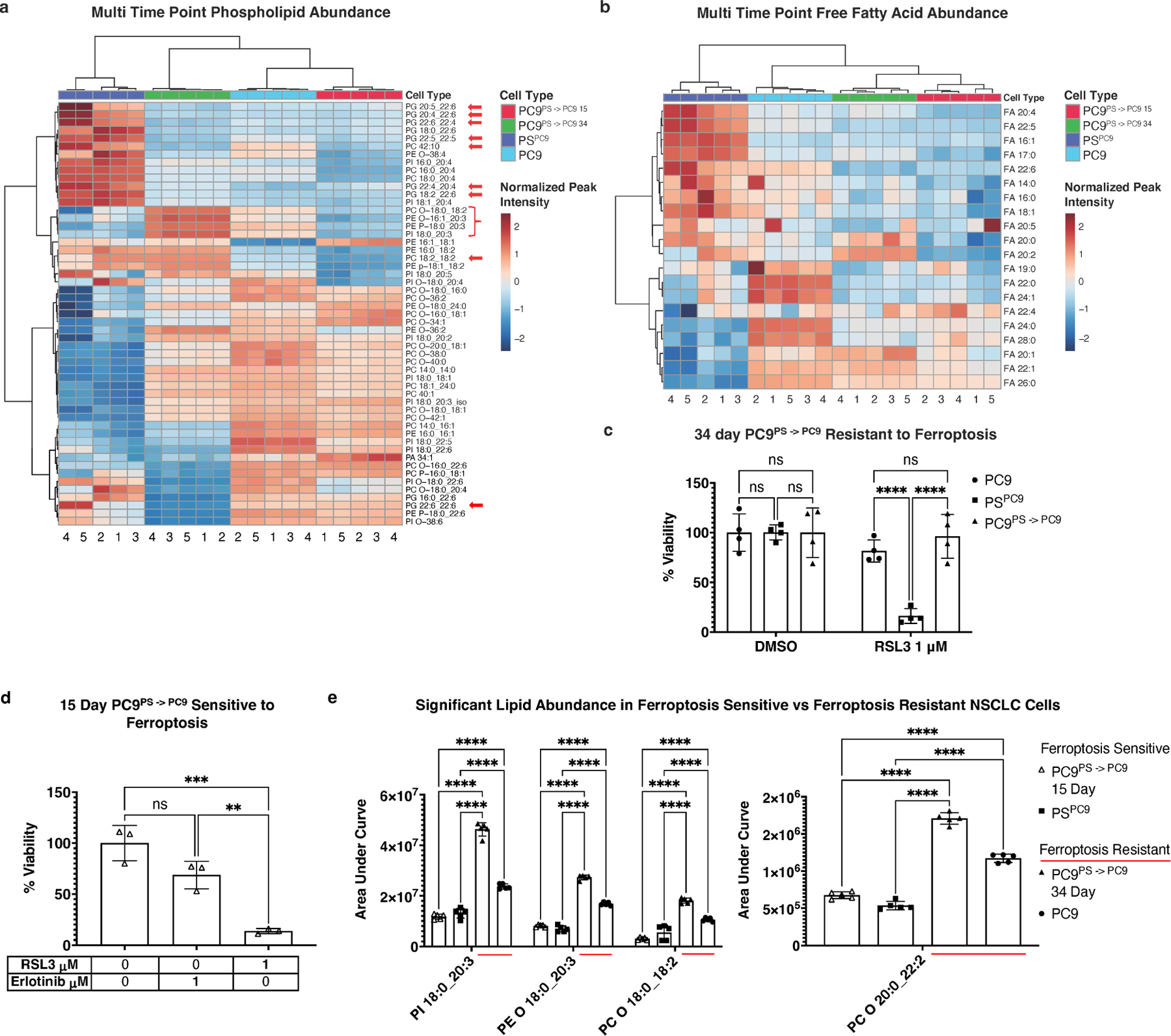
Untargeted lipidomic analysis of PC9/PS^PC9^ cells during PS reversion reveals re-acquisition of ferroptosis-resistant lipidome. (a) 53 of the 79 previously identified mono and PUFA-PLs; (b) 21 of the 26 previously identified FFA again identified among significantly (FDR < .05) changed abundance across all NSCLC cell types. Red arrows highlight diPUFA PLs. One-way ANOVA of normalized peak intensity with Euclidean distance and Ward clustering on rows and columns. 3 × 10^6^ cells per replicate sample for all cell types. Independently-cultured, erlotinib-treated biological replicates of PS cells cultured in sufficient quantity to pool into n = 5 replicates; PC9^PS –> PC9^ reverters derived from same batches of PS used in this experiment, independently cultured and expanded, and ultimately pooled into n = 5 replicates; n = 5 independently cultured biological replicates of PC9; (c) 48 h CTG viability assays of PC9, PS^PC9^, 34-day PC9^PS –> PC9^ and (d) 15-day PC9^PS –> PC9^, treated with 1 μM RSL3 or 1 μM erlotinib. Cells in viability assays were derived from the same batches used in lipidomics. n = 8 independently cultured, erlotinib-treated biological PS replicates, pooled into 4 replicates; n = 3 independently-cultured biological replicates of PC9 cells; (e) PUFA-PLs abundant and depleted in ferroptosis resistant (PC9, 34-day PC9^PS –> PC9^) vs sensitive (PS^PC9^, 15-day PC9^PS –> PC9^) cells, respectively. For (c) and (d), % viability relative to vehicle control, mean ± s.d, < 0.02% final in-well concentration of vehicle. In (e), data are presented as AUC of specific identified peaks, mean ± s.d; For (c-e), * P < 0.05, ** P < 0.01, *** P < 0.001, and **** P < 0.0001 by two-way or one-way ANOVA followed by Tukey’s multiple comparisons test. ns: non-significant; PUFA: polyunsaturated fatty acid phospholipids; PLs: phospholipids; FFA: free fatty acids; FDR: false discovery rate; NSCLC: non-small cell lung cancer; ANOVA: analysis of variance; CTG: CellTiter-Glo; RSL3: Ras Selective Lethal 3; AUC: area under the curve.

Strikingly, nearly all PUFA-PLs and FFA from the initial untargeted lipidomic analyses were again significant and differentially abundant (Figure 3a,b), with a similar lipid signature separating ferroptosis-sensitive PS^PC9^ and all other conditions (i.e., persister PS^PC9^ cells have elevated diPUFA-PLs and polyunsaturated FFA relative to PC9s, and also relative to both 15 day and 34 day reverting PC9^PS –> PC9^). Additionally, we observed reacquisition of ferroptosis resistance by 34 days (Figure 3c), and noted that globally, PS^PC9^ cells had a distinct lipid profile compared to parental PC9, 15 day PC9^PS –> PC9^, or 34 day PC9^PS –> PC9^ cells, all of which clustered together (Figure S3b).

These results were intriguing given that, although the previous (Figure 2) results were reproduced, the ferroptosis-sensitive 15 day PC9^PS –> PC9^ reverters (Figure 3d) had a lipid profile more similar to ferroptosis resistant PC9s and 34 day reverted PC9^PS –> PC9^ cells. We therefore examined other lipids that were differentially abundant between the ferroptosis-resistant PC9 cells and 34 day reverted PC9^PS –> PC9^ cells versus ferroptosis-sensitive PS^PC9^ and 15 day reverted PC9^PS –> PC9^ cells, which were still ferroptosis sensitive.

The importance of ether-linked PLs for mitochondrial ROS production and ferroptosis mitigation was noted by Chen *et al*.^[43]^, where a preponderance of MUFA and/or MUFA-tail-ether-linked lipids were detected in the mitochondria of PDX cells resistant to cell death induced by inhibiting mitochondrial complex I. We noted an increase in several mixed polyunsaturated-tail-containing ether-linked PLs in ferroptosis-resistant cells (Figure 3e, red underline).

Collectively, these findings indicate that in acquiring a ferroptosis-sensitive persister state, parental PC9 cells undergo a substantial shift in lipid architecture, resulting in a profile that is susceptible to ferroptosis. Upon exiting the persister state and re-acquiring ferroptosis resistance, 34 days reverted PC9^PS –> PC9^ cells ultimately regain a lipid architecture less conducive to ferroptosis and more similar to treatment-naïve PC9. Moreover, ferroptosis- driving diPUFA PLs are a key feature of this lipidomic remodeling. However, lipid abundance alone was not enough to determine ferroptosis sensitivity, and there are additional factors as indicated by the 15-day PC9^PS –> PC9^, at least in the PC9/PS^PC9^ cell model. We thus decided to test whether these lipidomics changes were similar in other persister cell models and identify the factors other than lipids that contribute to reacquisition of ferroptosis resistance.

### 3.5 PS features extend to prostate carcinoma (LNCaP) and fibrosarcoma (HT1080) persister models

**Figure 4.**
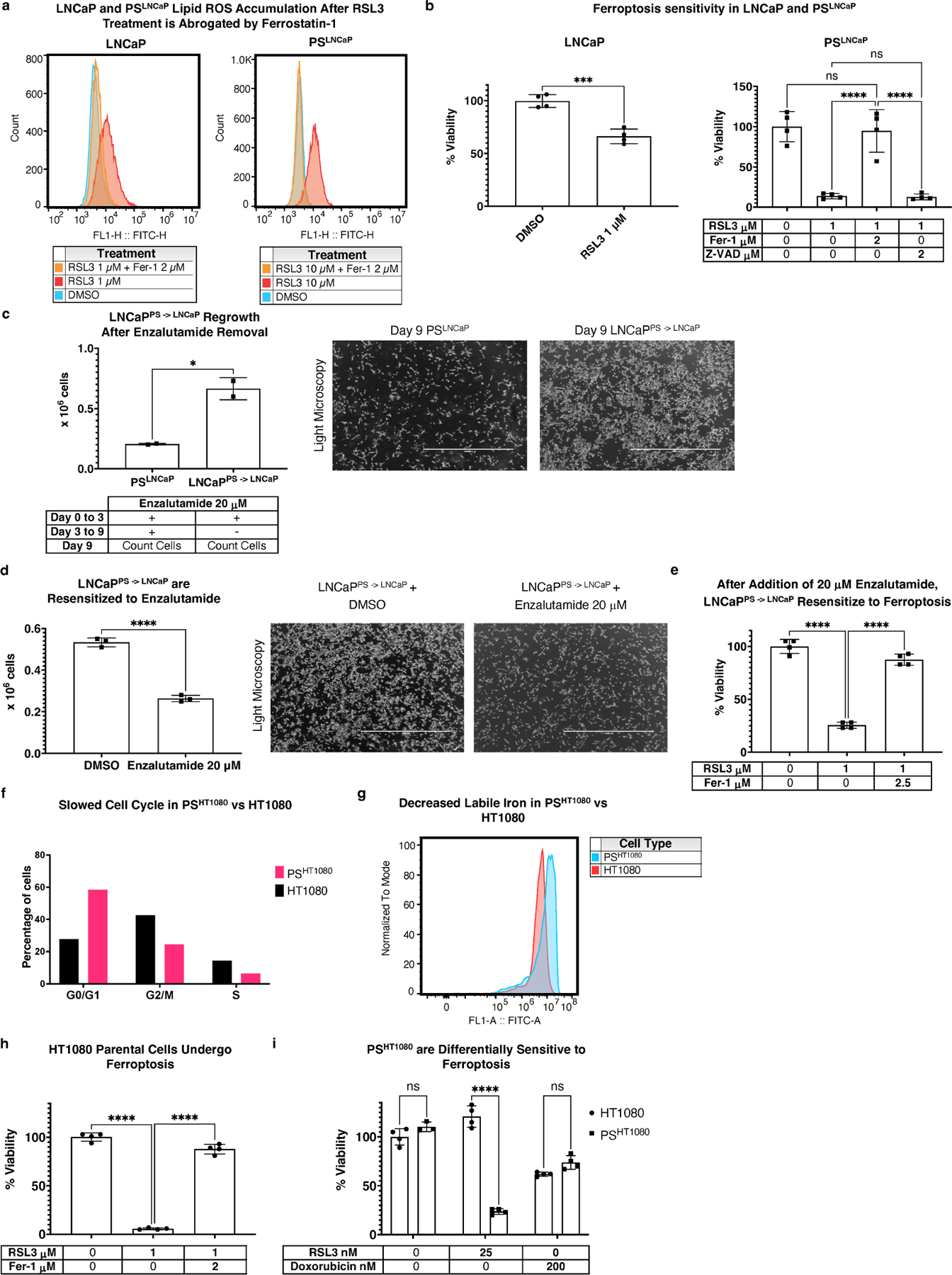
PS features extend to prostate carcinoma (LNCaP) and fibrosarcoma (HT1080) models. (a) Flow cytometry histogram of LNCaP and PS^LNCaP^ cells stained with C11-BODIPY for lipid ROS detection. Cells treated with DMSO, RSL3, and Fer-1; 1 or 10 μM RSL3 treatment, with or without 2 μM Fer-1. 2 × 10^4^ total events per condition; (b) 48 h CTG viability assay of LNCaP and PS^LNCaP^ cells treated with 1 μM RSL3, with and without 2 μM Fer-1 and 2 μM ZVAD. n = 8 independently cultured, enzalutamide-treated biological replicates of PS cells, pooled into 4 replicates; n = 3 independently cultured, biological replicates of PC9 cells; (c) Growth of re-derived LNCaP cells from persisters (LNCaP^PS –> LNCaP^) upon enzalutamide removal and (d) effect of 20 μM enzalutamide re-addition on LNCaP^PS –> LNCaP^ viability by trypan blue exclusion dye; (C) Enzalutamide removed day 3 of culture, cells counted day 9; for (d) 48 h of enzalutamide treatment. n = 2 (c) and n = 3 (d) independently cultured, biological replicates per experiment. Microscopy images from the same experiments used for viability for (c) and (d); 4x light microscopy of plates prior to viability assessment; (e) LNCaP^PS –>LNCaP^ subject to 2 day, 20 μM enzalutamide treatment, after which media was removed and cells treated with 1 μM RSL3, with and without 2.5 μM Fer-1 for 48 h, with CTG viability assay. n = 4 independently cultured, enzalutamide-treated biological replicates, pooled into 4 replicates; (f) Flow cytometry PI staining of HT1080 and PS^HT1080^ cells for percent of cells in each part of the cell cycle; 1 × 10^4^ events per sample; (g) Flow cytometry calcein-AM staining of HT1080 vs PS^HT1080^ cells for labile iron; 1 × 10^4^ events per sample; (h) 48 h CTG viability assay of HT1080 treated with 1 μM RSL3, with and without 2 μM Fer-1. n = 4 independently cultured biological replicates of HT1080; (i) 48 h CTG viability assay of HT1080 vs PS^HT1080^ cells, treated with 25 nM RSL3 or 200 nM doxorubicin. n = 8 independently cultured, doxorubicin-treated biological PS replicates, pooled into 4 replicates; n = 4 independently cultured biological replicates of HT1080; For (b, e, h and i), % viability relative to vehicle control, mean ± s.d, < 0.02% final in-well concentration of vehicle; * P < 0.05, ** P < 0.01, *** P < 0.001 and **** P < .0001 by one-way ANOVA followed by Tukey’s multiple comparisons test or unpaired two-tail t-test. ns: non-significant; ROS: reactive oxygen species; CTG: CellTiter-Glo; ANOVA: analysis of variance; RSL3: Ras Selective Lethal 3; PI: propidium iodide.

Recent work highlighted PS changes in LNCaP cells treated with enzalutamide, a chemotherapeutic agent for prostate carcinoma^[44]^. We therefore attempted to generate a PS-like state in LNCaPs by treating cells with enzalutamide for six days with a method similar to that used for the PC9/PS^PC9^ model. We observed that upon RSL3 treatment, lipid ROS were generated in both LNCaP and PS^LNCaP^ cells, and abrogated by the ferroptosis inhibitor ferrostatin-1 (Fer-1) (Figure 4a), indicating that both LNCaPs and PS^LNCaP^ cells undergo ferroptosis. However, when both LNCaP and PS^LNCaP^ were treated with the same concentration of RSL3, PS^LNCaP^ viability dropped to around 10% whereas LNCaP remained at around 60%; PS^LNCaP^ cell death was not rescued by Z-VAD (Figure 4b).

Upon enzalutamide removal from the medium after three days and subsequent culture for a total of 9 days, LNCaP^PS –> LNCaP^ cells resumed rapid proliferation relative to PS^LNCaP^ cells (Figure 4c), and regained enzalutamide sensitivity when added back into the media of proliferating LNCaP^PS –> LNCaP^ cells (Figure 4d). Strikingly, when reverted LNCaP^PS –> LNCaP^ cells were again cultured with enzalutamide in PS generating conditions, they again were sensitive to RSL3-induced ferroptosis (Figure 4e).

Collectively, these data indicate that the PS^LNCaP^ cells are: (1) more sensitive to ferroptosis versus LNCaP, (2) reversible by enzalutimide removal, resulting in LNCaP^PS –> LNCaP^ reversion, (3) re-sensitized to enzalutamide again after LNCaP^PS –> LNCaP^ expansion, and (4) re-sensitized to ferroptosis again, upon addition of enzalutamide to LNCaP^PS –> LNCaP^. These findings expand on previous work^[44]^, and suggest that enzalutamide treatment enriches for PS cells in LNCaP cell cultures.

Given the observations using a relevant agent in LNCaPs, and the work of Schwab *et al*.^[21]^ using doxorubicin in U2OS osteosarcoma cells, we tested doxorubicin in HT1080 fibrosacroma cells for its ability to generate PS^HT1080^ as above—6 days of doxorubicin culture. We found that the cell cycle slowed in the six day PS^HT1080^ cells (Figure 4f), and cells no longer required passaging (typical HT1080 cells require splitting every 2 days). Additionally, Hangauer *et al*.^[3]^ demonstrated that for PC9/PS^PC9^ cells, labile iron levels are reduced in PS^PC9^. We found this to be true for PS^HT1080^ vs HT1080 as well (Figure 4g). It is established that HT1080 cells are sensitive to ferroptosis, and we confirmed that the HT1080s used in this model are sensitive as expected (Figure 4h). However, we found that after six-day doxorubicin culture, collecting, evaluating viability, and re-seeding and treating with RSL3, PS^HT1080^ cells were sensitized to RSL3-induced ferroptosis vs HT1080s, even beyond the normal sensitivity (Figure 4i).

Collectively, these observations (slowed cell cycle, increased labile iron, and drug-induced ferroptosis sensitization) are consistent with a PS-like phenotype for PS^HT1080^ cells. Therefore, given these results for LNCaP and HT1080 PS cells, we assessed whether the PC9/PS^PC9^ lipidomic findings were recapitulated in these additional PS-like systems.

### 3.6 Untargeted lipidomics of LNCaP/PS^LNCaP^ and HT1080/PS^HT1080^ models reveals altered lipid profiles, and addition of diPUFA-PLs augments ferroptosis sensitivity

**Figure 5.**
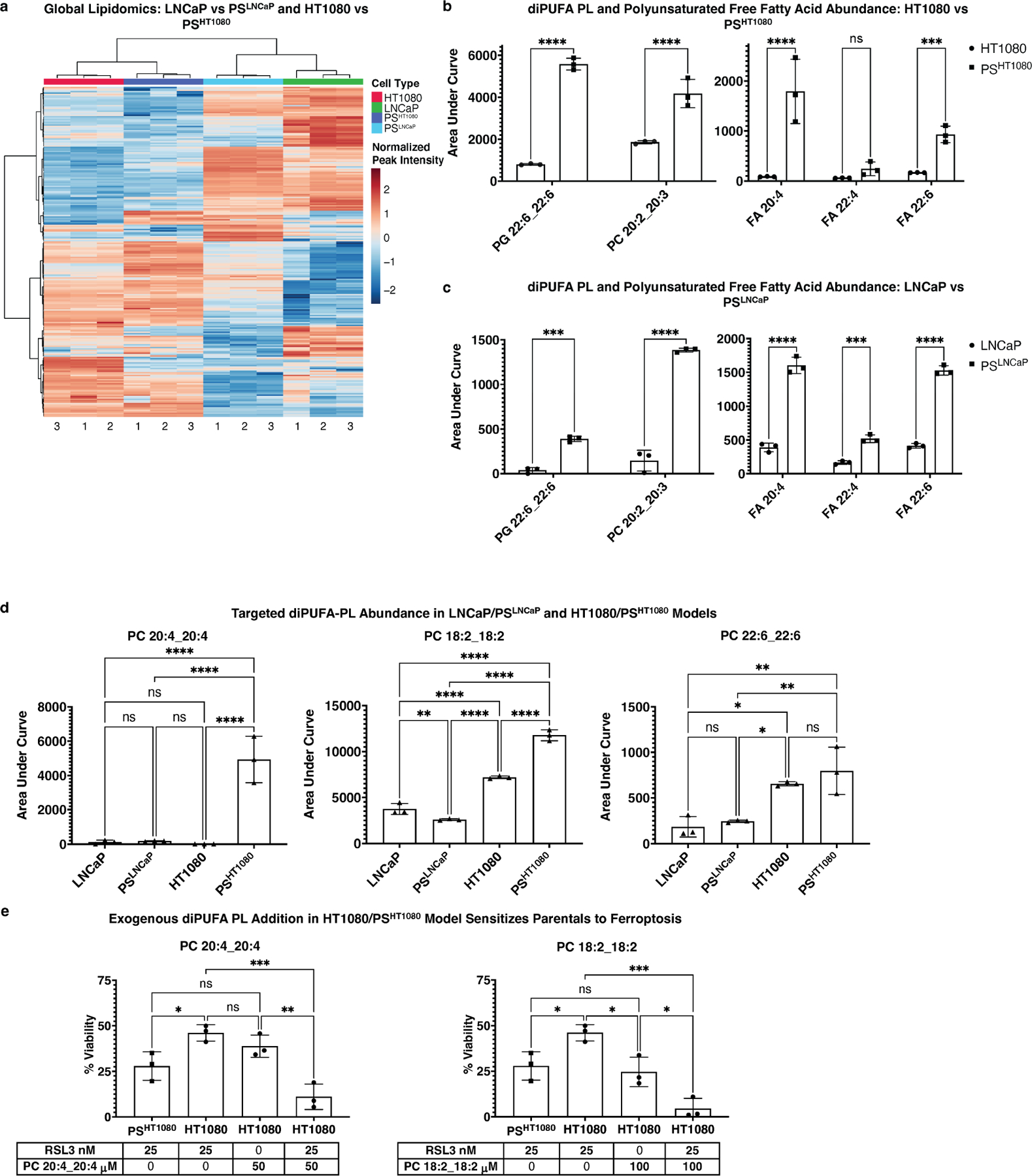
Untargeted lipidomics of LNCaP/PS^LNCaP^ and HT1080/PS^HT1080^ models reveals a mixed lipid profile, and addition of diPUFA-PLs augments ferroptosis sensitivity. (a) Clustering profile of all significant (FDR < .05) detected m/z RT values for LNCaP, PS^LNCaP^, HT1080, and PS^HT1080^. 3 × 10^6^ cells per sample for all cell types. One-way ANOVA of normalized peak intensity with Euclidean distance and Ward clustering on rows and columns. Independently cultured, enzalutamide treated (for LNCaP) or doxorubicin treated (for HT1080) biological replicates of PS cultured in sufficient quantity to pool into n = 3 replicates; n = 3 independently cultured biological replicates of LNCaP or HT1080; (b) Comparison of specific identified diPUFA-PL and polyunsaturated FFA peak intensities from untargeted lipidomics in fibrosarcoma and (c) prostate carcinoma models; (d) Targeted diPUFA-PL abundance in prostate carcinoma and fibrosarcoma persister models; retention time and m/z validated by standards run in tandem; (e) 24 h CTG viability assay of HT1080 and PS^HT1080^ cells treated with 25 nM RSL3 to induce ferroptosis after sensitization with exogenous PC(20:4_20:4) at 50 μM and PC(18:2_18:2) at 100 μM. n = 8 independently cultured, doxorubicin-treated biological replicates of PS, pooled into 3 replicates; n = 3 independently cultured, biological replicates of HT1080; For (b-d), data are presented as AUC of specific identified peaks, mean ± s.d; For (e), % viability relative to vehicle control, mean ± s.d, < 0.1% final in-well concentration of vehicle; For (b-e), * P < 0.05, ** P < 0.01, *** P < 0.001, and **** P < 0.0001 by two-way or one-way ANOVA followed by Tukey’s multiple comparisons test. ns: non-significant; diPUFA: di-polyunsaturated fatty acid phospholipids; PLs: phospholipids; ANOVA: analysis of variance; AUC: area under the curve.

In light of our lipidomic findings in the PC9/PS^PC9^ cell model, we performed untargeted lipidomics on HT1080 fibrosarcoma and LNCaP prostate carcinoma PS-like cell models. Globally, cells clustered by tissue of origin (Figure 5a), regardless of persister state. We identified several diPUFA-PL and polyunsaturated FFA elevated in PS vs parental cells across these tissue types (Figure 5b,c), notably including PG(22:6_22:6), a diPUFA PL previously shown to be a key effector of ferroptosis sensitivity^[45]^. However, we did not detect other diPUFA PLs as enriched, similar to what was observed in PS^PC9^ cells vs PC9 cells. Hence, the specific lipidomic changes vary in different cell models.

We performed a targeted approach and examined diPUFA lipid standards by LC-MS to confirm M/Z values and RT, including those shown to drive ferroptosis sensitivity (e.g., PC(20:4_20:4). We observed that PC(20:4_20:4) was significant and with a large effect size overabundant in PS^HT1080^ vs HT1080 cells, but not in the LNCaP model (Figure 5d). Similarly, PC(18:2_18:2) was significant and with a large effect size overabundant in PS^HT1080^ vs HT1080 cells, but not in the LNCaP model. PC(22:6_22:6) was more abundant in the HT1080 PS model vs the LNCaP PS model, yet was not differentially abundant in either of PS-like cells vs their parental counterparts.

Exogenous addition of PUFAs sensitizes cells to ferroptosis^[20,46]^; when diPUFA-PLs identified as differentially abundant in PS^HT1080^ persisters (e.g., PC(18:2_18:2)) were added to parental HT1080 cells, they were sensitized to ferroptosis to at least the level of the PS^HT1080^ cells, if not more (Figure 5e).

Together, these data illustrate the changing lipid profile across cell models. The elevation of diPUFA-PLs in PS-like cells and the addition of exogenous diPUFA-PLs sensitizing parental cells to the level of their persister counterparts, suggests that increased diPUFA lipids are one factor contributing to the increased sensitivity of persister cells to ferroptosis, but other lipidomic changes in each model could also contribute, and that non-lipidomic factors may also play a role.

We therefore focused on assessing whether the ferroptosis sensitivity of PS cells could be reversed, and whether such reversion could provide an insight into what causes PS ferroptosis sensitivity.

### 3.7 Mitochondrial elimination in PS^HT1080^ cells partially reverts ferroptosis sensitivity and alters the lipid profile

Given that diPUFA-PLs initiate ferroptosis through the mitochondria^[20]^, and that sublethal miniMOMP was demonstrated to be an upstream driver of persister state formation in PC9/PS^PC9^ model^[17]^, we interrogated whether the presence of mitochondria could drive PS ferroptosis sensitivity.

It has previously been demonstrated in parkin-expressing HT-1080 cells that using carbonyl cyanide m-chlorophenyl hydrazone (CCCP) induces subsequent parkin based mitochondrial elimination (i.e., mitophagy)^[34]^. Specifically, this method was shown to: (1) not affect GPX4 protein levels, (2) not affect ferroptosis sensitivity in wild-type (i.e., non-parkin expressing) HT1080 cells, (3) not affect RSL3 sensitivity in an off-target manner (i.e., effects observed were the effect of mitochondrial elimination and not CCCP itself), (4) deplete multiple mitochondria markers (e.g., tom20, cytochrome c, complex I), (5) prevent mitochondria-dependent apoptosis, and (6) not affect cell viability.

**Figure 6.**
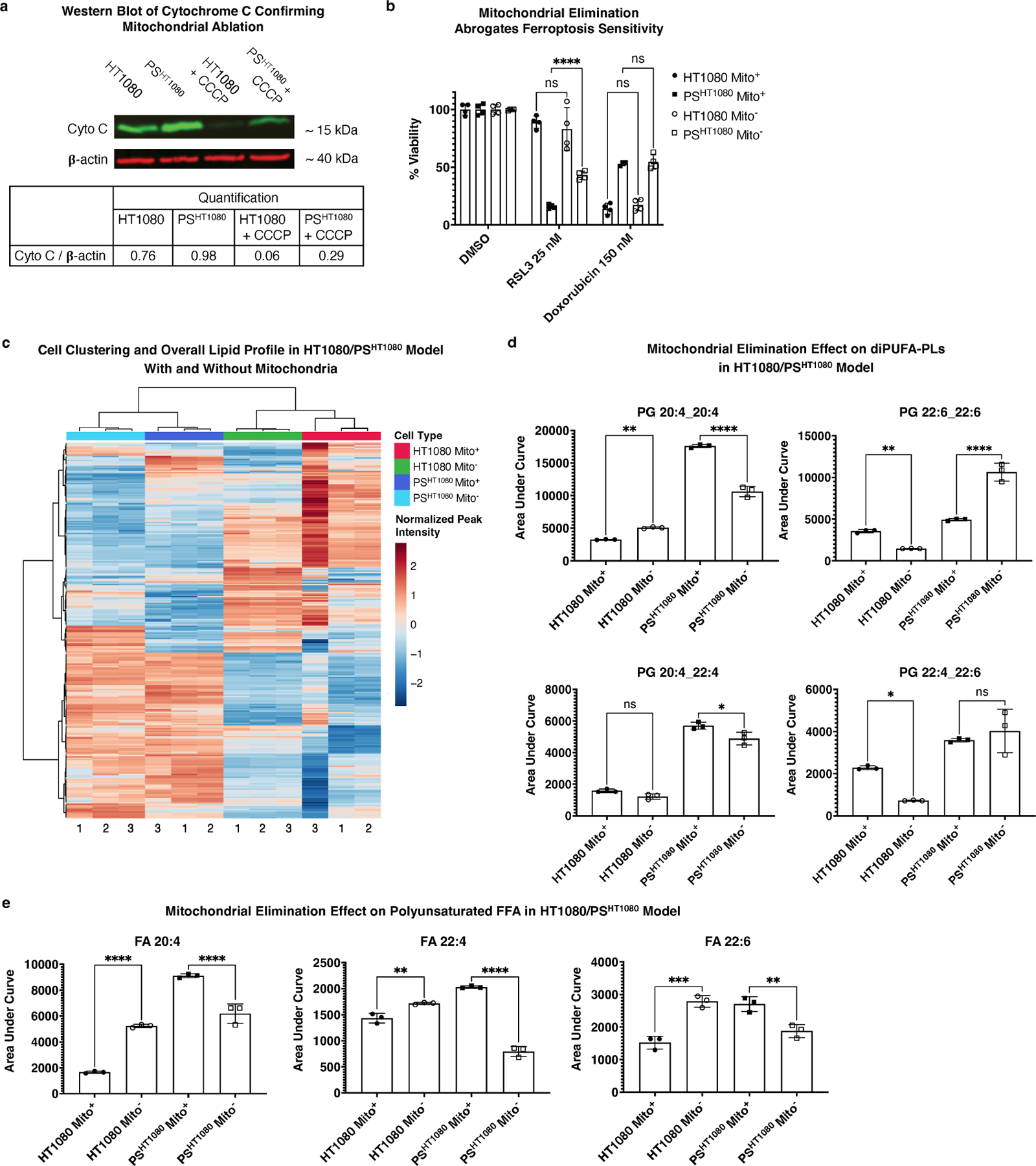
Mitochondrial elimination in PS^HT1080^ reverts PS ferroptosis sensitivity and alters the lipid profile. (a) Western blot of Cytochrome C (~15 kDa) with β-actin (~40 kDa) as control of parkin expressing HT1080 (Mito^+^) vs PS^HT1080^ (Mito^+^) vs HT1080 + CCCP (Mito^−^) vs PS^HT1080^ + CCCP (Mito^−^) and (b) 56hr CTG viability assay of 25 nM RSL3 or 150 nM doxorubicin treated fibrosarcoma cells under same conditions; CCCP is a mitochondrial uncoupling agent that in cells expressing parkin protein results in mitochondrial elimination as previously described^[34]^. n = 8 and n = 12 independently cultured, doxorubicin treated biological replicates of PS for Mito^+^ and Mito^−^, respectively, pooled into n = 4 replicates; n = 4 independently cultured biological replicates of HT1080; (a) and (b) are from same batches of cells for all conditions; (c) Clustering of all significant (FDR < .05) detected m/z RT values for fibrosarcoma model with and without mitochondria. 3 × 10^6^ cells per sample for all cell types. One-way ANOVA of normalized peak intensity with Euclidean distance and Ward clustering on rows and columns. Independently cultured, doxorubicin treated biological replicates of PS cultured in sufficient quantity to pool into n = 3 replicates; n = 3 independently cultured biological replicates HT1080 Mito^+^ and Mito^−^; (d) Mitochondrial ablation effect in fibrosarcoma model on specific diPUFA-PLs and e, polyunsaturated FFAs; For (b) % viability relative to vehicle control, mean ± s.d, < 0.02% final in-well concentration of vehicle; For (d) and (e), data are presented as AUC of specific identified peaks, mean ± s.d., For (b-e), * P < 0.05, ** P < 0.01, *** P < 0.001, and **** P < 0.001 by two-way ANOVA followed by Šidák’s or Tukey’s multiple comparisons test. ns: non-significant; CCCP: carbonyl cyanide m-chlorophenyl hydrazone; FDR: false discovery rate; RT: retention times; ANOVA: analysis of variance; diPUFA: di-polyunsaturated fatty acid phospholipids; PLs: phospholipids.

We used this method to eliminate mitochondria in the parkin-expressing HT1080s cells as described above, and six-day doxorubicin-treated parkin-expressing PS^HT1080^ cells derived from those parental cells. This resulted in abrogation of RSL3-induced ferroptosis sensitivity in the PS^HT1080^ from the previously observed approxumately23% viability in cells with mitochondria (Figure 4i) to around 54% in cells without (Figure S4a,b). We then performed a head-to-head viability assay between HT1080 and PS^HT1080^ cells, with and without mitochondria (HT1080 Mito^±^, PS^HT1080^ Mito^±^). Upon mitochondrial elimination, we observed abrogation of ferroptosis sensitivity in PS^HT1080^ Mito^−^ (from around16% viability to 43%), but no change was observed in ferroptosis sensitivity for HT1080 Mito^−^ or to doxorubicin for any of the cells (Figure 6a,b). These re-capitulated previous observations that mitochondrial elimination does not affect RSL3 ferroptosis sensitivity in HT1080 cells^[34]^.

We then used LC-MS to interrogate a direct connection between the presence of mitochondria and a potential ferroptosis-conducive lipid profile (i.e., as in that of the PC9/PS^PC9^ system). Across all significant m/z RT values, cells clustered according to PS status rather than mitochondrial presence (Figure 6c). Among specifically identified diPUFA-PLs, mitochondria-eliminated PS^HT1080^ Mito^−^ had decreased levels of diPUFA-PLs (e.g., PG(20:4_20:4)) (Figure 6d) andpolyunsaturated FFA (e.g., 20:4, 22:4, and 20:6) (Figure 6e). We also noted that some diPUFA-PL levels were not changed (e.g., PG(22:4_22:6) or even increased in the ferroptosis-resistant Mito^−^ persister state (e.g., PG(22:6_22:6)) (Figure 6d), despite being elevated in the ferroptosis-sensitive PS^HT1080^ Mito^+^ cells relative to HT1080 parental cells.

Collectively, these data indicate that mitochondria play a key role in maintaining ferroptosis sensitivity in PS^HT1080^ cells, as mitochondrial elimination partially abrogates ferroptosis sensitivity. Although mitochondrial elimination did affect the PS cell lipid architecture, it was altered inconsistently: some pro-ferroptotic diPUFA-PLs and polyunsaturated FFAs were reduced, while other ferroptosis-promoting lipids were unchanged.

### 3.8 PS ferroptosis-relevant protein signatures vary across cell models, and ATF4 elimination does not abrogate ferroptosis sensitivity in PS^PC9^ cells

ATF4 plays a key role downstream of mini-MOMP in PS^PC9^ formation and maintenance and is a known modulator of ferroptosis in non-PS contexts. Additionally, Zhang *et al*. reported that GPX4 is overexpressed in two colorectal cancer (CRC) cell lines 5-fluorouracil induced PS^[47]^. In the PC9/PS^PC9^ model, ATF4 expression was elevated in PS^PC9^ vs PC9 (Figure S5a), which is consistent with previous observations^[17]^, but with respect to GPX4, we observed the opposite expression pattern from Zhang *et al*. PC9 persister cells exhibited depletion of GPX4 (and also the ferroptosis inhibitor GCH1) (Figure S5b,c), both of which reverted upon PC9^PS–>PC9^ rederivation, along with ATF4 (Figure S5d). We also observed in the LNCaP/PS^LNCaP^ model, equal levels of GPX4 and ATF4 between PS and parental cells (Figure S5e). Moreover, in the HT1080/PS^HT1080^ model, we observed ATF4 expression levels in opposition to that observed in PC9/PS^PC9^ cells (Figure S5f). Notably, neither short term siRNA nor long term CRISPR-Cas9 ATF4 elimination abrogated ferroptosis sensitivity in PS^PC9^ (Figure S6).

In the context of PS cells as a model for minimal residual disease, we attempted to generate a mouse model to assess the feasibility of targeting PS cells *in vivo*, but were unsuccessful (Figure S7). We verified that the PC9 cells used for xenografting generated PS in vitro (Figure S7a), and injected into the flanks of immunodeficient nude mice the cells that came from that same batch of PC9 cells. Mice were treated for 10 days with vehicle, erlotinib, or the novel GPX4 inhibitor VP224, a compound developed in our lab with improved ADME properties relative to RSL3 (Figure S7b). These treatments were followed by a 2 day “drug holiday” mimicking the minimal residual disease state, and then a subsequent 10 days treatment with either vehicle, erlotinib, or the VP224, in combinations that would target PS cells had they developed during the initial 10 day treatment course (Figure S7c). However, IHC staining for PS^PC9^ specific markers CD24 and CD133 was equivocal between erlotinib, VP224, and vehicle treated tumors (Figure S7d), and no persister cells were detectable in vivo.

## 4. Discussion

This work explored multiple aspects of PS ferroptosis sensitivity, both in terms of acquisition and maintenance. Our data support the presence of mitochondria as a key factor for maintenance of ferroptosis sensitivity once the persister state is achieved. We found that the PS^PC9^ lipid profile is primed to undergo ferroptosis via enrichment of diPUFA PLs, as well as polyunsaturated FFA. Additionally, upon re-derivation of parental cells from persisters and re-acquisition of ferroptosis resistance, the PC9^PS –> PC9^ lipid profile reverted to a parental-like lipid architecture. Moreover, exogenous addition of diPUFA lipids alone was sufficient to sensitize HT1080 cells to the degree equal to (or more) of ferroptosis sensitivity seen in PS-like PS^HT1080^, and mitochondrial ablation substantially and consistently (though not fully) abrogated PS^HT1080^ ferroptosis sensitivity. Thus, in complement with previous work demonstrating that exogenous addition of diPUFA PLs triggers ferroptosis via accumulation in mitochondria^[20]^, our findings implicate mitochondria as at least partial architects of ferroptosis sensitivity in the PS context.

Although direct elimination of mitochondria from HT1080/PS^HT1080^ cells resulted in lipid profile changes and depletion of key polyunsaturated FFA and diPUFA-PL species in persister cells, some diPUFA lipids remained abundant or unchanged. This is in line with the fact that mitochondrial elimination accounts for only partial ferroptosis sensitivity reversion suggesting other lipid remodeling elements outside the mitochondria are important. CCCP treatment is known to have various off target effects, and though the use of this parkin-based system and its effects on multiple aspects of HT1080 cell apoptosis and ferroptosis sensitivity have been described^[34]^, it is possible that off target effects exist in PS^HT1080^ cells that do not exist in HT1080. Moreover, an important highlight of these mitophagy results is that they complement non-persister context work previously observed: both Gaschler *et al*.^[34]^ and Mao *et al*.^[48]^ demonstrated that mitochondrial ablation does not abrogate HT1080 ferroptosis sensitivity under GPX4 inhibition, although Mao *et al*.^[48]^ did show mitochondrial regulation of cysteine starvation induced ferroptosis. Combined with the mitochondrial localization patterns of exogenously added diPUFA observed by Qiu *et al*.^[20]^ in a PS independent model, our results suggest that a key metabolic shift occurs within PSs in the underpinnings of the ferroptosis susceptibility mechanism itself, and not simply an increased susceptibility resulting from the same but now improved drug targeting mechanism (e.g., higher binding affinity). Whether this is a consequence of prolonged prevention of oncogenic signaling, chromatin remodeling, both, or something entirely different remains to be explored. A critical next step would be to use an orthogonal method of mitochondrial elimination as validation, and ideally these systems would be transferred into PC9/PS^PC9^ cells, a well-established system in PS research. In conjunction, since previous exogenous lipid addition and subsequent ferroptosis sensitization^[20]^ was 1) not performed in a PS context and 2) did not use the exact diPUFA-PLs used in this work, an important subsequent confirmation would be to perform exogenous lipid addition experiment with ferroptosis specific rescue agents, ensuring that this is indeed ferroptosis being induced, and not an alternative form of cell death.

We were intrigued to observe an increase in diPUFA-PLs abundance in PS^PC9^ cells, a finding recapitulated across two independent untargeted lipidomics experiments conducted months apart, and to see the signature (by 34 days) reverted in accordance with ferroptosis sensitivity. However, given the 15-day PC9^PS –> PC9^ observation, though lipid profile changes do occur, these changes alone cannot fully explain the whole picture.

The diPUFA-PL signatures in a targeted analysis of HT1080 and LNCaP vs PS-like cell models showed a varied profile, some were significantly abundant in PSs, whereas others showed an opposite signature, and some were not changed. One lipid, PG(22:6_22:6), was significantly attenuated in abundance between two independent lipidomics experiments, though it remained abundant in PS-like cells (HT1080 and LNCaP vs PS in Figure 5 and the HT1080 vs PS^HT1080^ with and without mitochondria in Figure 6). Abundance of a single, non-exogenously added diPUFA, despite its implication in other ferroptosis-related diseases (e.g., Huntington vs control human brain tissue^[20]^) is unlikely to determine the entire sensitivity profile. Globally, HT1080 and LNCaP cells vs respective PS cells clustered by tissue type rather than PS status. Moreover, according to the transcriptomic data, one of the single strongest individual gene effects was decreased SCD in PS^PC9^ relative to PC9, which could facilitate some of the observed changes in lipid architecture as well. Collectively, this adds weight to the idea that lipid abundance and type, though clearly important to ferroptosis, are not the sole drivers of ferroptosis sensitivity in PS. One can postulate, for example, the following scenario: PC9 cells undergo lipid profile and protein expression changes by 6 days in becoming PS^PC9^, but during reversion (PC9^PS –> PC9^), protein levels take longer to return to pre-chemotherapeutic treatment levels. Alternatively, and not mutually exclusively, given that the PS state and its reversion are centered around chromatin remodeling, different suites of proteins could be involved in the to-PS transition vs the from-PS transition.

Ferroptosis sensitivity is likely driven by several factors, and we did observe mitigation of ferroptosis sensitivity by mitochondrial elimination. It seems likely that there is an interplay between lipid architecture, mitochondrial presence, and ferroptosis sensitivity during the acquisition of the persister state and reversion back to parental cells. Our studies provide several snapshots of this dynamic process. Moreover, the lipidomic analyses highlight the importance of looking at persister systems from different tissues of origin through the same analytical lens.

Recent work by Schwab *et al*.^[21]^ demonstrated the importance of transcription factor ZEB1 in lipid membrane remodeling and ferroptosis sensitivity across multiple cancer models, including bronchioalveolar carcinoma, pancreatic adenocarcinoma, and osteosarcoma derived with similar methods as the PS used here. Although the focus was not on diPUFA-PLs or factors around the PS state and its reversal, evidence demonstrated the increase of monoPUFA phospholipids in ferroptosis-sensitive cells in a ZEB1-dependent manner, that in consistent with our observations. However, their noted near absence of oxidized phospholipids in cells depleted of ZEB1 (and thus resistant to ferroptosis) was also observed, which was not mirrored in our models, highlighting the complexity of lipid architecture, protein expression, and its interplay with ferroptosis sensitivity across multiple systems.

We did explore the role of several ferroptosis-modulating proteins but found the results to be inconclusive. It is plausible that different tissues of origin could have the same key players in persister state formation and maintenance, but these factors might be differently modulated depending on other features of that particular tissue/cancer cell model. Additionally, while not explicitly performed in a PS system, findings by Liang^[49]^ and colleagues demonstrated that MBOAT2 suppression leads to phospholipid profile remodeling and ferroptosis sensitization, which is consistent with our findings in PS cells. It would be valuable to explore which aspects of ferroptosis sensitization, if any, are specific to LNCaP and HT1080s (the two cell lines used in both our and Liang *et al*.^[49]^ study) vs PS^LNCaP^ and PS^HT1080^, and how these aspects might manifest through time in the attainment and maintenance of the persister state.

Finally, it should be noted that even with all of the above taken into consideration, PS ferroptosis sensitivity has only been demonstrated as correlative to date: it has not been disproven that the observed persister sensitization to ferroptosis does not arise independently from acquisition of the persister state (e.g., downregulation of MBOAT2 leads to lipid architecture remodeling and thus causes ferroptosis sensitization, but MBOAT2 downregulation is purely an effect of the therapeutic, and is not the cause of PS formation). Similarly, findings by Kalkavan *et al*.^[17]^ regarding the generation of PC9- derived PS through sublethal, incomplete-MOMP are illuminating, both with respect to the mechanisms of PS formation and the sensitization of these induced PS to ferroptosis. Specifically, cells that were deficient in ATF4 and HRI, upon surviving BH3 mimetic treatment, were not as sensitized to ferroptosis. However, this presents a catch-22 situation: these PC9 cells were deficient in ATF4 and HRI to begin with, and thus presumably, cannot become true PS, so whatever abrogation of ferroptosis sensitivity occurs is exactly independent of persister state acquisition—these cells lack the very tools necessary to become PSs, at least as far as is known. Though the PS-like HT1080 system is novel and less can be said about potential mechanisms of signaling in the context of further ferroptosis sensitization and PS-like state formation, a similar argument can be made: Prolonged exposure to doxorubicin may induce a PS-like state and enhance ferroptosis sensitivity, but these two effects need not be causally linked. Indeed, PS formation—the winding and unwinding of particular sections of chromatin in order to persist in the face of chemotherapeutics—does not necessarily need to be causal to PS ferroptosis sensitization in general, and even if it is, the sensitization may merely be a “passenger” feature, i.e., even upon full restoration of ferroptosis resistance, PS would still continue to persist under drug treatment.

Our attempt at using PC9 cells to generate persisters *in vivo* using erlotinib in a model of minimal residual disease did offer insights for future directions. The model likely failed to generate persister cells in the erlotinib-treated group due to a short time course—at 10 days, there was no appreciable difference in tumor volume between the 3 groups, and thus no reason to think one group had become enriched for persisters due to treatment, which would explain lack of substantial difference between treatment arms in the second half of the experiment. However, by approximately 20 days, the exclusive vehicle treatment arm gained a substantial increase in tumor volume, and the treated mice in the other groups survived to the end of the trial—this may represent a promising time course shift for future experiments, i.e., doing a 20-day initial treatment with erlotinib to generate a persister state, and then performing the second phase of the trial with a ferroptosis inducer.

This study has several limitations. Firstly, in terms of mechanistic insight, the findings regarding diPUFA-PLs in the PC9/PS^PC9^ system are both intriguing and reproducible, and the effect of mitochondrial elimination on PS-like PS^HT1080^ cells suggest a potential mechanistic link. Another limitation is that the lipidomics findings are not fully consistent across LNCaP and HT1080 systems, and mitochondrial elimination did not result in the type of diPUFA-PL enrichment that was observed in PS^PC9^. Finally, the PS^HT1080^ system is a newer system. Though the ability to take a relevant chemotherapeutic agent for a particular cancer, add the agent in culture with those cancers immortalized cell line, and produce cells with at least some PS-like characteristics (as was also done with U2OS^[21]^) is encouraging, this system can only be called PS-like, and cannot be used to make substantial conclusions regarding modulators of ferroptosis sensitivity in more established PS systems.

## 5. Conclusion

All PS and PS-like cells used in this study, regardless of tissue type or chemotherapeutic agent used, exhibited similar phenotypes of ferroptosis sensitivity and arrest of cell cycle. This suggests a unifying role of ferroptosis susceptibility to maintenance of the persister state, and highlights the need for further investigation into the relationship between chemotherapeutic resistance and ferroptosis susceptibility; of course, drug resistance is not synonymous with the persister state, as cancer cells can be drug resistant without being persisters, although persisters are generally drug resistant. Moreover, persister cells may simply experience prolonged inhibition of signaling from cancer drug treatments, that ultimately leads to altered lipid profiles. In other words, the mechanism driving the formation of persister cells remains unclear in many models. In addition, we observed intriguing differences (protein expression, lipid abundance, etc.) across cancer tissue types, underlying unifying features of ferroptosis sensitivity. These differences emphasize the importance of studying multiple PS models in order to understand the mechanistic drivers underlying features which, may superficially appear identical across tissues. This becomes especially important in a clinical setting, where individualized approaches to ferroptosis induction (or prevention) may become necessary depending on the cancer tissue of origin and disease state. Thus, the mechanistic aspects we have explored could eventually be leveraged as a tool against minimal residual disease across multiple cancer types, potentially complimenting and enhancing other treatments in the prevention of cancer recurrence.

## Supplementary Material

Supplementary Materials

Supplementary materials

The supplementary material for this article is available at: Supplementary material.

## Data Availability

The data supporting the findings of this study are available within the paper and its supplementary information. RNA-seq data are available in the NCBI’s GEO database under the accession code GSE300857. Lipidomics data is deposited in Metabolights under accession code MTBLS12980. All data reported in this paper will be shared by the Lead Contact upon request. Any additional information required to reanalyze the data reported is available from the Lead Contact upon request.
