# Supplementary material for: Lipidomic changes in persister cancer cells drive enhanced ferroptosis sensitivity
Source: Ferroptosis Oxid Stress. Author manuscript; Available in PMC 2025 Dec 26. (PMC12739762; doi:10.70401/fos.2025.0003)
Supplement: Supplementary Materials [file NIHMS2122980-supplement-Supplementary_Materials.pdf]

Ferroptosis and Oxidative Stress
https://doi.org/10.70401/fos.2025.0003

Supplementary information

Lipidomic changes in persister cancer cells drive enhanced ferroptosis sensitivity

Eduard Reznik, Fereshteh Zandkarimi, Joleen M. Csuka, Qiulin Zhu, Jenny Jin, Taruna Vani Neelakantan, Jiewen Zheng, Vasiliki Polychronidou, Mark Fongheiser, Ashley Brown, Baiyu Qiu, Megan Rodriguez, Lela DeVine, Prem S Subramaniam, Wei Gu, Andrea Califano, Brent R. Stockwell

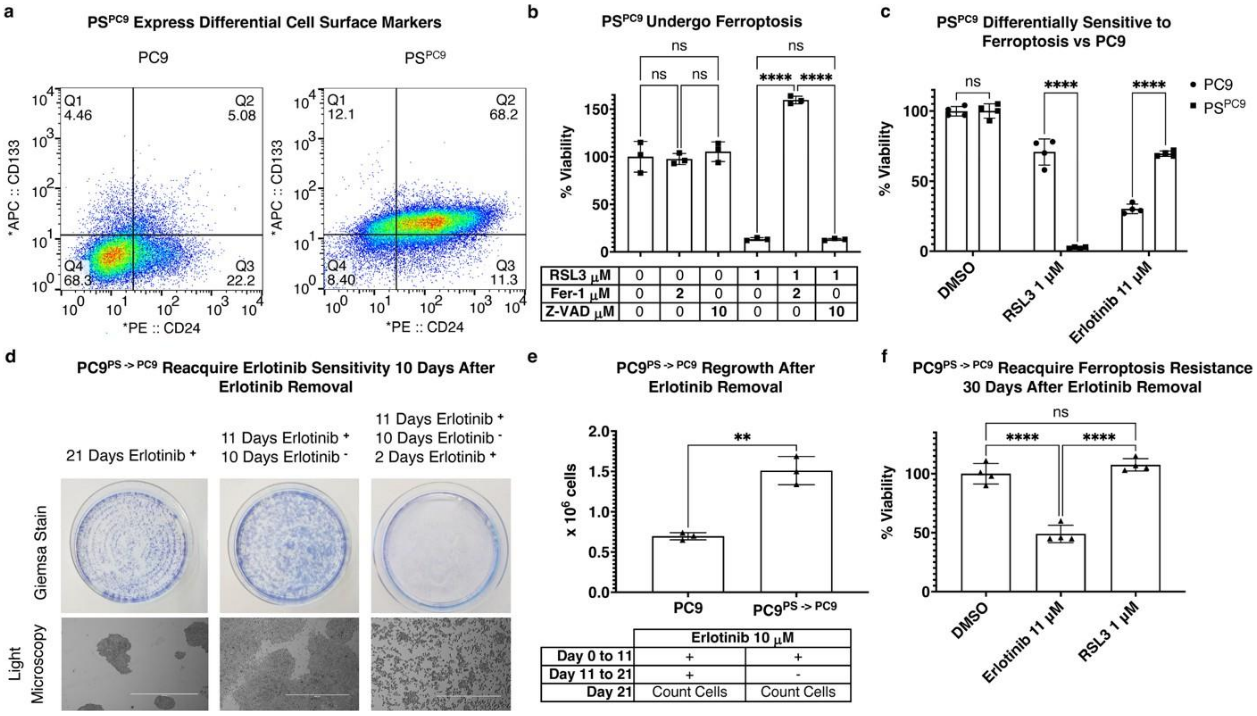

Figure S1. 6-day PC9/PS^{PC9} model has persister features. (a) PC9 and PS^{PC9} cells stained for CD24, CD133, and sorted by FACS. 1x10^5 events per cell type; (b) 48 hr CTG viability assay of PS^{PC9} cells treated with 1 μM RSL3 with and without 2 μM ferrostatin-1 (Fer-1) or 10 μM Z-VAD-FMK (ZVAD) and (c) PS^{PC9} vs PC9 cells treated with 1 μM RSL3 or 11 μM erlotinib. *n* = 8 independently cultured, erlotinib-treated biological PS cell replicates (8 for b and 8 for c, respectively), pooled into *n* = 3 replicates for (b) and n = 4 replicates for (c); *n* = 4 independently cultured biological replicates for PC9 cells; (d) Giemsa stain and corresponding 4x light microscopy images of PS^{PC9} cells grown in 10 μM erlotinib (left), with subsequent removal of erlotinib and regrowth (center), and re-introduction of 10 μM erlotinib (right); (e) Cell count of viable rederived PC9$^{PS->PC9}$ upon erlotinib removal and subsequent growth/expansion by trypan blue exclusion dye; erlotinib removed day 11, cells counted day 21. *n* = 8 independently cultured, erlotinib treated biological PS replicates, pooled into *n* = 3 replicates; *n* = 3 independently cultured PC9$^{PS->PC9}$; (f) 48 hr CTG viability assay of PC9$^{PS -> PC9}$ cells 30 days post 10 μM erlotinib removal, treated with 11 μM erlotinib or 1 μM RSL3. *n* = 4 independently cultured, biological PC9$^{PS->PC9}$ replicates. Microscopy images (d), regrowth (e), and re-aquistion of ferroptosis resistance and erlotinib sensitivity (f) are from same batches of cells. For (b, c, and f) % viability relative to vehicle control, mean ± s.d, < 0.02% final in-well concentration of vehicle; * *P* < 0.05, ** *P* < 0.01, *** *P* < 0.001, and **** *P* < 0.0001 by two-way or one-way ANOVA followed by Tukey's multiple comparisons test or unpaired two- tail t-test. ns = non-significant.

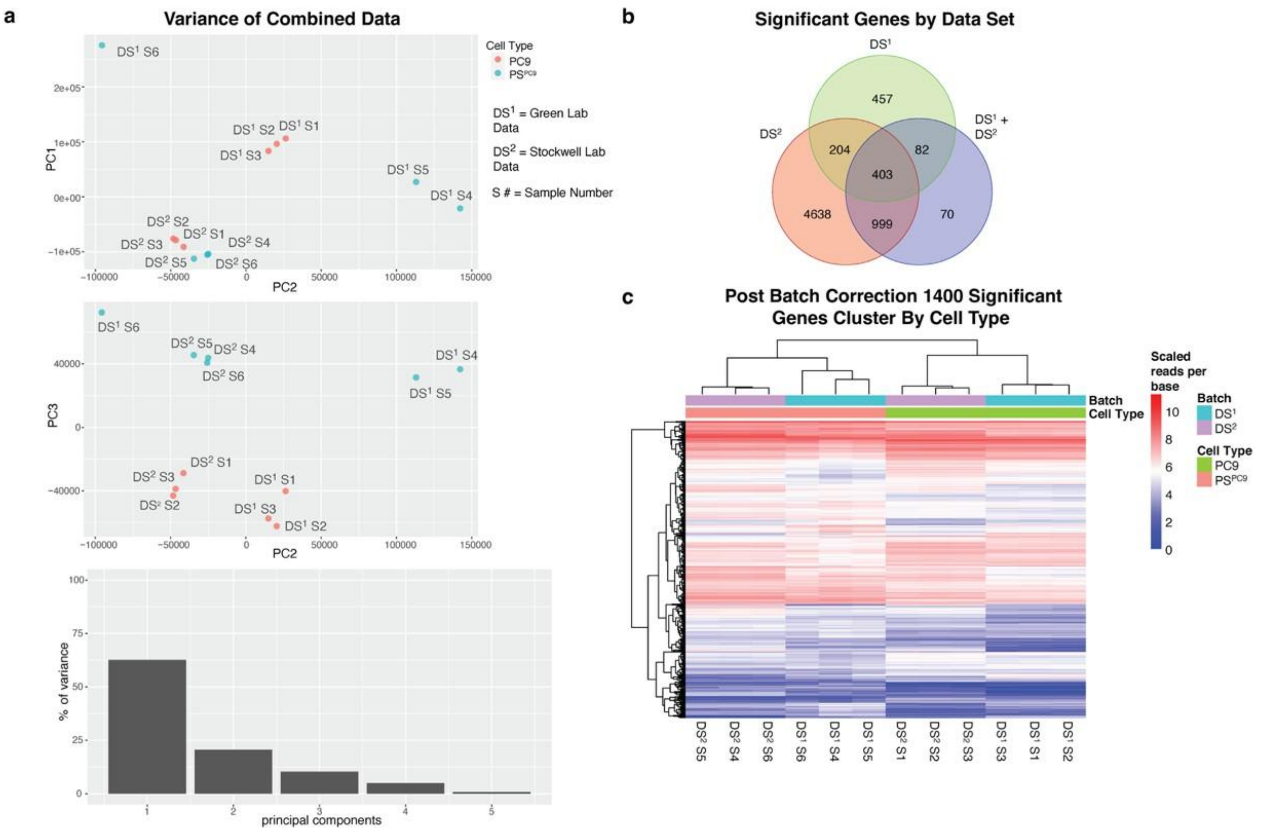

Figure S2. Identification and removal of batch effect in RNAseq data. (a) Principal component (PC) 1 vs PC 2, PC 2 vs PC 3, and % variance accounted for by each PC of Green (DS^1) and Stockwell (DS^2) lab samples prior to batch correction (top, middle, and bottom, respectively); (b) Intersection of significant (FDR < .01) differentially expressed genes (DEG) by data set (DS^1, DS^2, and $DS^1 + DS^2$) and c, clustering on ~1400 significant DEG post batch correction. Sample statistics same as Figure 1.

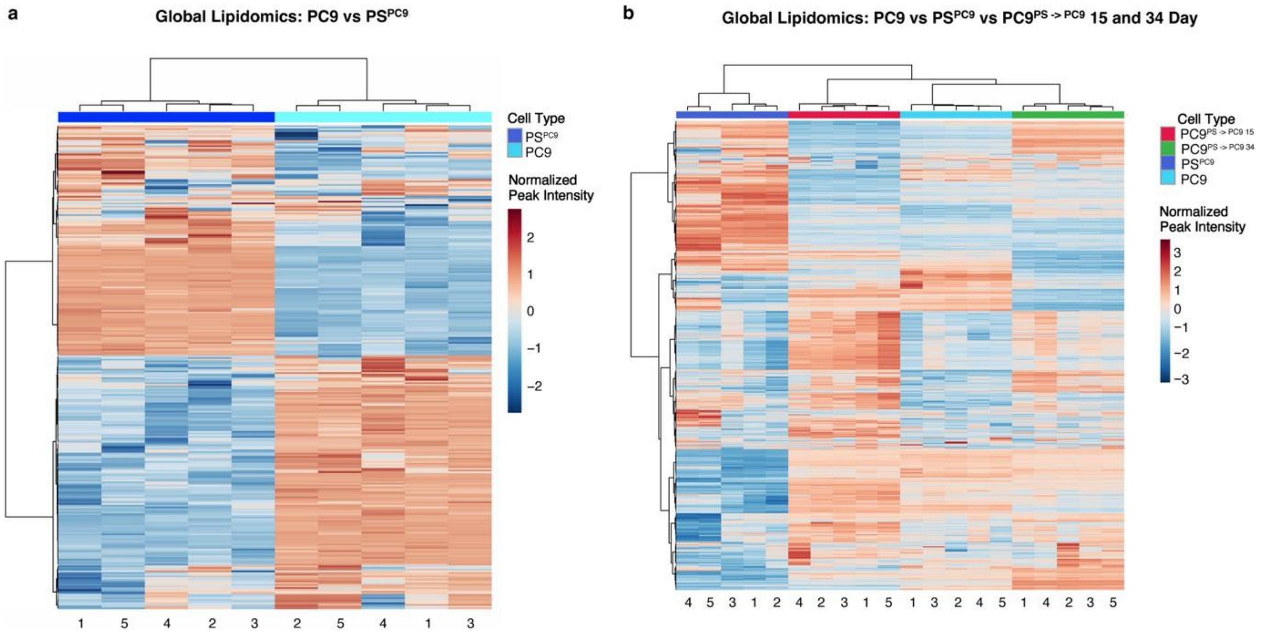

Figure S3. Global lipidomic profile of PC9/PS^{PC9} cells. Clustering profile of all detected m/z RT values for (a) PC9 vs PS^{PC9} and (b) PC9 vs PS vs 15 and 34 day $PC9^{PS \to PC9}$. One-way ANOVA of normalized peak intensity with Euclidean distance and Ward clustering on rows and columns. Sample statistics for (a) and (b) same as Figure 2 and Figure 3, respectively.

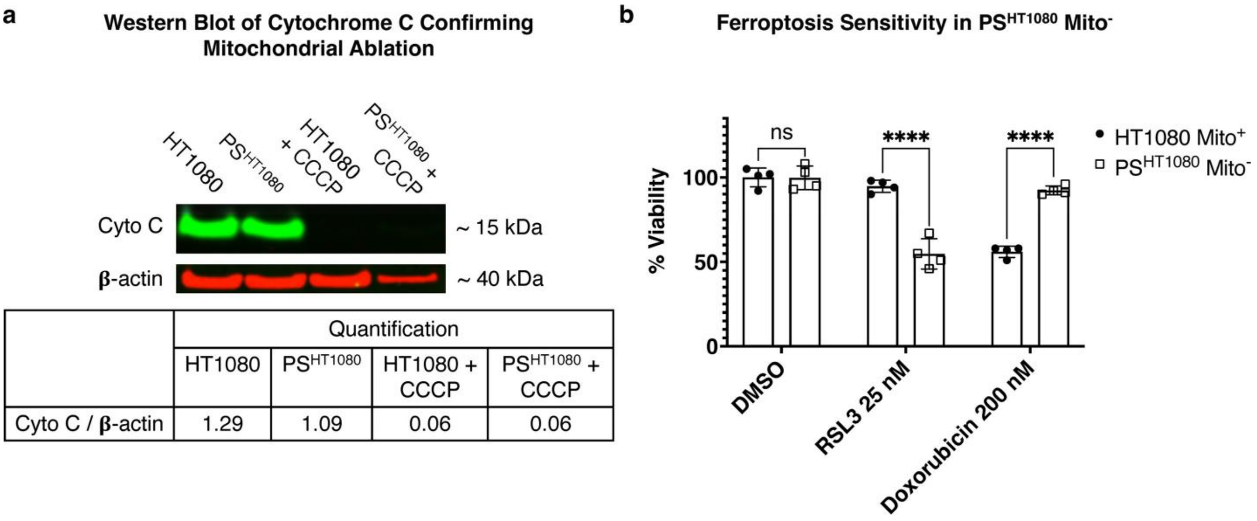

Figure S4. Mitochondrial elimination in PS^{HT1080} partially reverts ferroptosis sensitivity. (a) Western blot of cytochrome C with ß-actin as control of parkin-expressing HT1080 (Mito^{+}) vs PS^{HT1080} (Mito^{+}) vs HT1080 + CCCP (Mito^{-}) vs PS^{HT1080} + CCCP (Mito^{-}) and **b,** 48 hr CTG viability assay of HT1080 Mito^{+} vs PS^{HT1080} Mito^{-} treated with 25 nM RSL3 or 200 nM doxorubicin. *n* = 12 independently cultured, doxorubicin-treated biological PS replicates, pooled into *n* = 4 replicates; *n* = 4 independently cultured, biological replicates of HT1080; (a) and (b) are from same batches of cells for HT1080 Mito^{+} and PS^{HT1080} Mito^{-} conditions. Data are percent viability relative to vehicle control, mean ± s.d, < 0.02% final in-well concentration of vehicle; * P < 0.05, ** P < 0.01, *** P < 0.001, and **** P < 0.001 by two-way ANOVA followed by Tukey's multiple comparisons test. ns = non-significant.

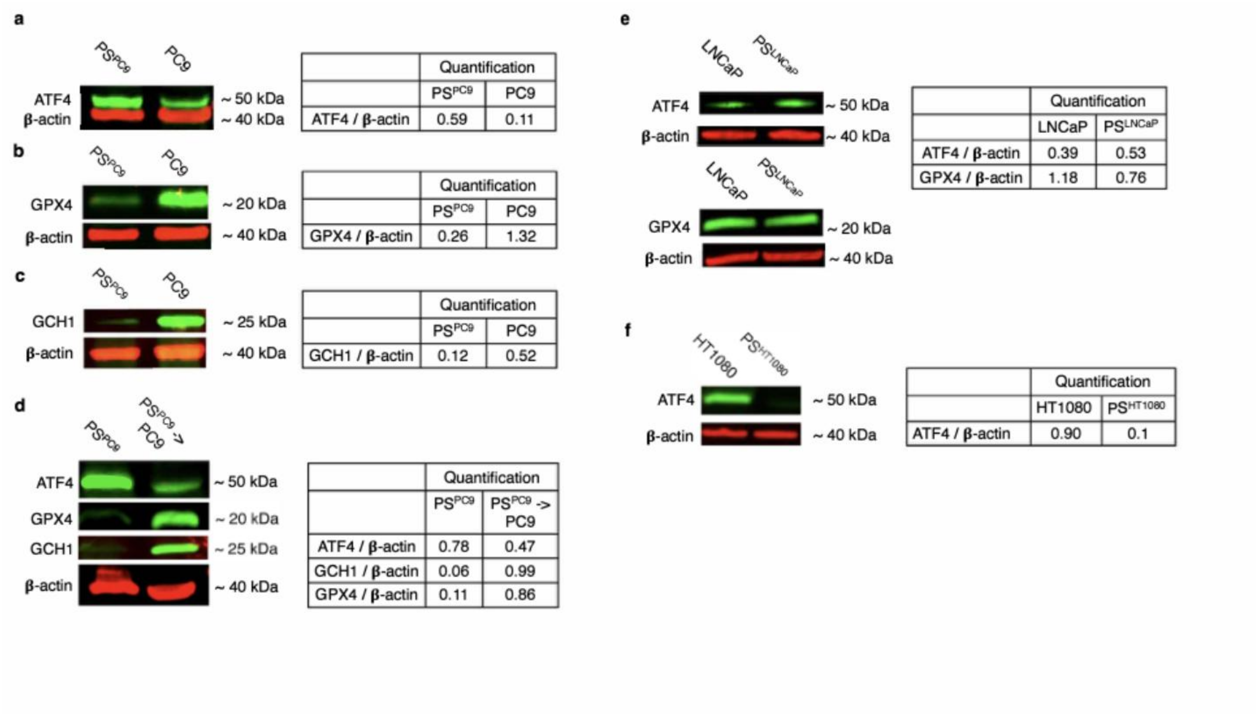

Figure S5. PS and ferroptosis relevant protein signatures vary across cell models. Western blot of PS^{PC9} and PC9 cells stained for (a) ATF4 (~50kDa); (b) GPX4 (~20kDa), and (c) GCH1 (~25 kDa) with � actin (~40 kDa) as control for all; (d) ATF4, GPX4, and GCH1 � actin control stain in direct comparison of PS^{PC9} and PC9$^{PS\ ->\ PC9}$. *n* = 8 independently cultured, erlotinib treated biological PS replicates, pooled into replicate per blot; *n* = 3 independently cultured biological replicates for PC9; *n* = 2 independently cultured, biological PC9$^{PS\ -}$

$^{> PC9}$ replicates, pooled into replicate per blot; (e) Western blot of LNCaP and PS^{LNCaP} for ATF4 and GPX4, with �-actin as control. n = 8 (for PS^{LNCaP}) independently cultured, enzalutamide-treated biological PS replicates, pooled into replicate per blot; n = 2 independently cultured, biological replicates for LNCaP; (f) ATF4, with ß-actin as control of HT1080 vs PS^{HT1080}. n = 8 independently cultured, doxorubicin treated biological PS replicates, pooled into replicate per blot; n = 2 independently cultured, biological replicates of HT1080, pooled into replicate per blot.

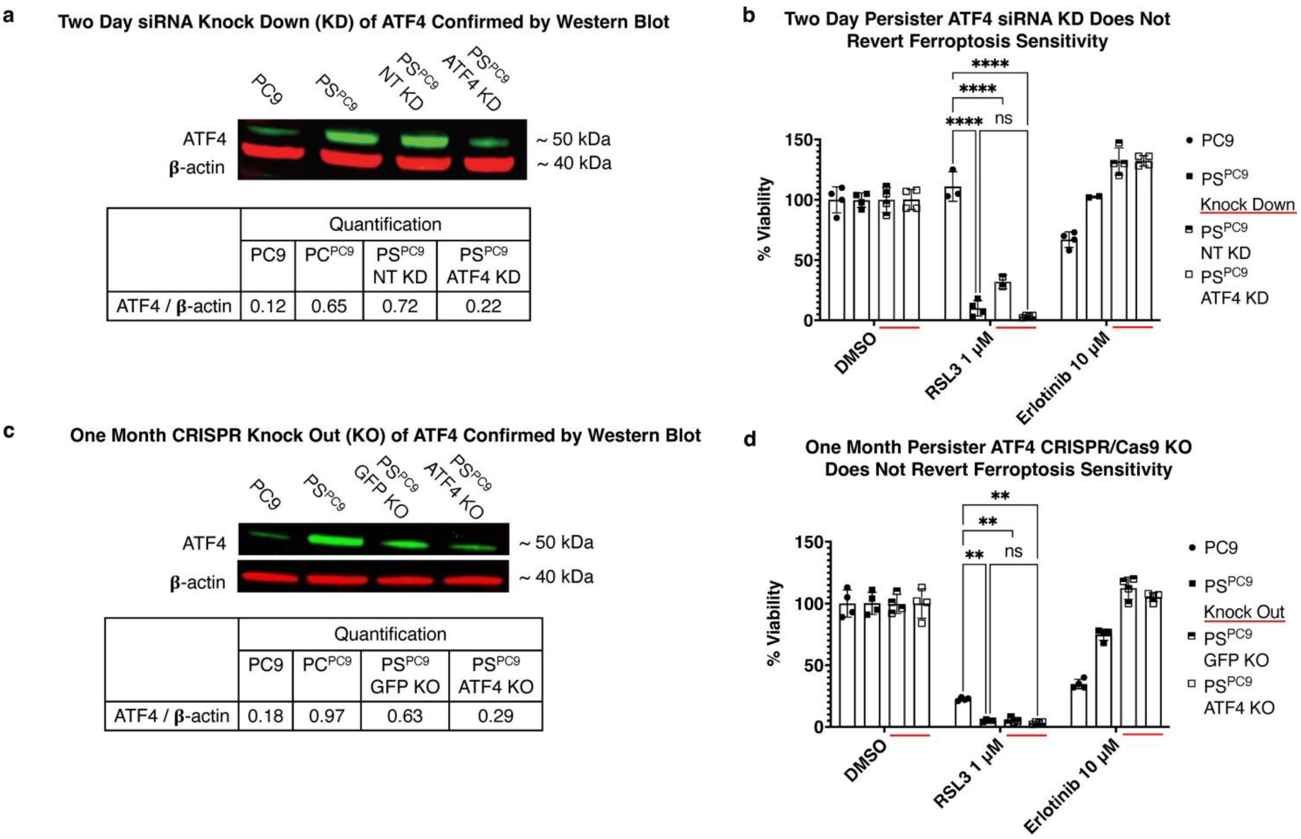

	Quantification			
	PC9	PC^{PC9}	PS^{PC9} NT KD	PS^{PC9} ATF4 KD
ATF4 / β-actin	0.12	0.65	0.72	0.22

	Quantification			
	PC9	PC^{PC9}	PS^{PC9} GFP KO	PS^{PC9} ATF4 KO
ATF4 / β-actin	0.18	0.97	0.63	0.29

Figure S6. ATF4 elimination did not abrogate ferroptosis sensitivity in PS^{PC9}. (a) Western blot for ATF4 with �-actin control in PC9 vs PS^{PC9} vs PS^{PC9} non-targeting siRNA (NT KD) vs PS^{PC9} ATF4 targeting siRNA (ATF4 KD) and (b) 48hr CTG viability assay of those same cells treated with 1 μM RSL3 or 10 μM erlotinib; NT KD = control; KD length = 2 days; n = 8, n = 12, and n = 12 independently cultured, erlotinib treated biological PS replicates for PS^{PC9}, PS^{PC9} NT KD, and PS^{PC9} ATF4 KD, respectively, pooled into n = 4 replicates for viability and replicate per blot for western; n = 4 independently cultured biological replicates for PC9, a portion of each sampled and pooled into replicate per blot for western; (c) Western blot for ATF4 with �-actin control in PC9 vs PS^{PC9} vs PS^{PC9} GFP-targeting sgRNA (GFP KO) vs PS^{PC9} ATF4-targeting sgRNA (ATF4 KO) and d, 48hr CTG viability assay of KO cells treated with 1 μM RSL3 or 10 μM erlotinib; GFP KO = control; KO length = 1 month; sample statistics/size identical to siRNA KD. For (b) and (d) % viability relative to vehicle control, mean ± s.d, < 0.02% final in-well concentration of vehicle; * $P < 0.05$, ** $P < 0.01$, *** $P < 0.001$, and **** $P < 0.001$ by two-way ANOVA followed by Tukey's multiple comparisons test.

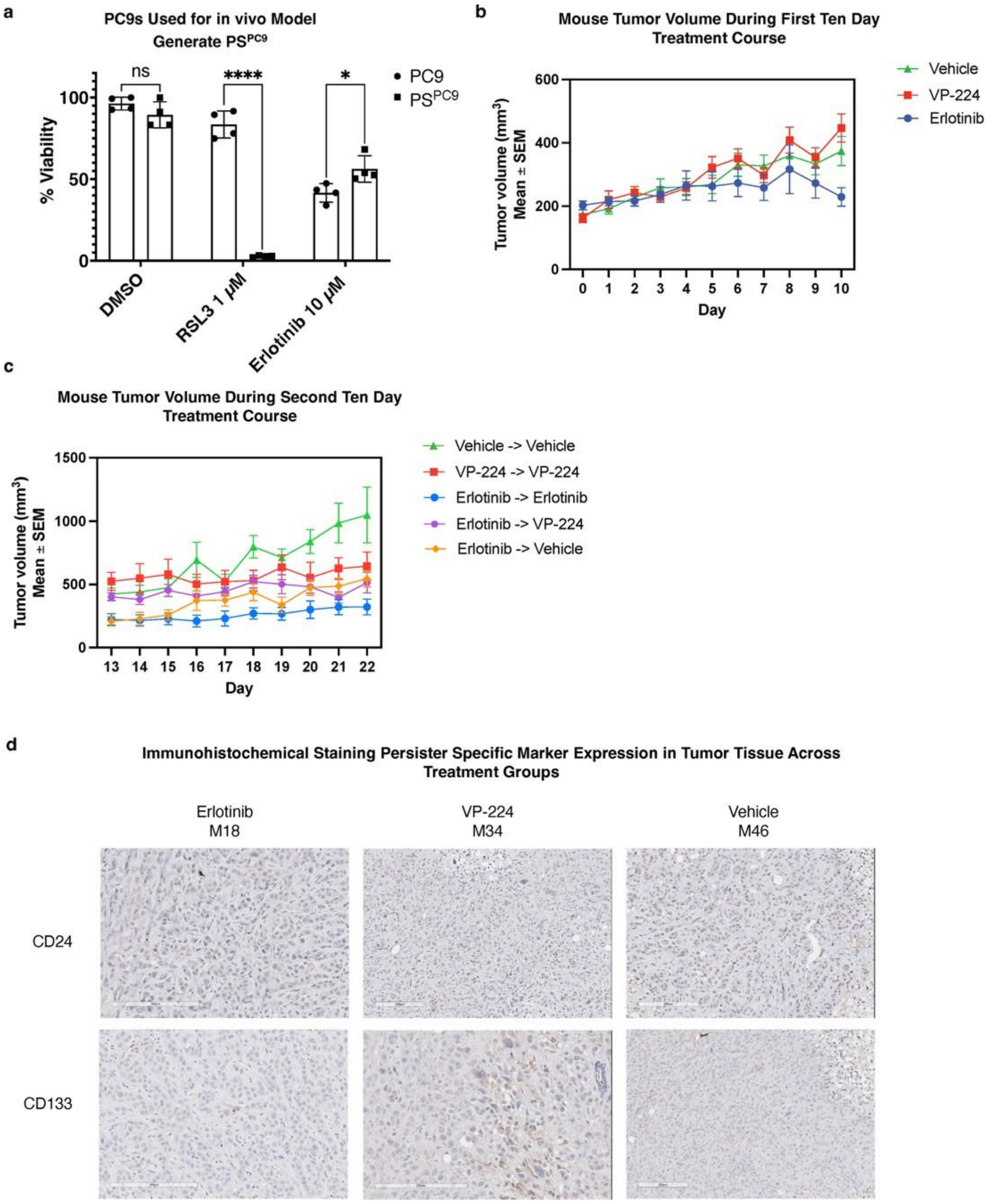

Figure S7. PS^{PC9} cells were not generated *in vivo*. (a) 48 hr CTG viability assay of PS^{PC9} vs PC9 treated with RSL3. n = 8 independently cultured, erlotinib treated biological PS replicates, pooled into *n* = 4 replicates; n = 4 independently cultured replicates for PC9; (b) Effect on tumor volume of vehicle, erlotinib, or VP-224 treatment during first and (c), second ten-day trial period; compound preceding "->" indicates treatment during initial ten days, compound post "->" indicates treatment after the two day drug holiday, for final ten day period; 5×10^6 cells from same batch of PC9 used in (a) injected sub-cutaneous into right flank to generate tumors; treatment initiated once average tumor volume per group was 200 mm^3; (d) Representative images showing immunohistochemistry staining of CD24 and CD133 expression (persister markers) in erlotininb, VP-224, and vehicle treated tumor tissue, bar = 200 μm. M# indicates the mouse ID tumor was taken from. For (a), % viability relative to vehicle control, mean ± s.d., * $P < 0.05$, ** $P < 0.01$, *** $P < 0.001$, and **** $P < 0.001$ by two- way ANOVA followed by Tukey's multiple comparisons test. ns = non-significant. For (b) and (c), data are presented as tumor volume per group, mean ± s.d. Statistics by nested one-way ANOVA summarized in source data.

Table S1. First page of table demonstrating gene rank changes of pre and post-batch effect corrected RNAseq data. Target_id is the ensemble gene identifier. Rank_naive is the rank of the gene by significance pre-batch effect correction, after model fitting. Rank_corrected is the rank of the gene by significance post-batch effect correction, after model fitting. Relative_difference in position shift calculated as absolute_value(rank_naive − rank_corrected)/(rank_naive + rank_corrected). Showing first page of 330 rank shifted genes with initial naïve q_val < .05. Sample statistics same as **Figure 1**.

target_id	qval_corrected	rank_corrected	qval_naive	rank_naive	relative_difference
ENSG00000128422	2.01E-07	1	9.89E-05	4	6.00E-01
ENSG00000125966	2.28E-07	2	2.29E-05	1	3.33E-01
ENSG00000198074	3.80E-07	3	7.46E-05	2	2.00E-01
ENSG00000128965	1.46E-06	4	5.18E-03	64	8.82E-01
ENSG00000176907	1.90E-06	5	1.91E-03	27	6.88E-01
ENSG00000173391	2.02E-06	7	1.13E-03	17	4.17E-01
ENSG00000056558	2.02E-06	8	6.29E-03	78	8.14E-01
ENSG00000147852	2.02E-06	9	7.19E-04	9	0.00E+00
ENSG00000099194	2.57E-06	12	9.89E-05	3	6.00E-01
ENSG00000163283	3.27E-06	13	1.13E-03	18	1.61E-01
ENSG00000169174	3.49E-06	14	3.49E-04	5	4.74E-01
ENSG00000134013	3.96E-06	15	2.49E-03	34	3.88E-01
ENSG00000176532	3.96E-06	18	3.95E-03	51	4.78E-01
ENSG00000134824	5.65E-06	19	1.59E-03	22	7.32E-02
ENSG00000109814	7.17E-06	20	1.81E-03	24	9.09E-02
ENSG00000113739	7.90E-06	22	4.21E-02	280	8.54E-01
ENSG00000185567	8.40E-06	23	3.85E-02	248	8.30E-01
ENSG00000023839	8.40E-06	24	1.72E-02	154	7.30E-01
ENSG00000184012	9.30E-06	25	1.03E-03	14	2.82E-01
ENSG00000198910	9.30E-06	26	4.50E-02	299	8.40E-01
ENSG00000182197	9.30E-06	27	7.00E-03	85	5.18E-01
ENSG00000135636	9.30E-06	28	3.42E-03	43	2.11E-01
ENSG00000116717	9.30E-06	29	1.42E-02	139	6.55E-01
ENSG00000008517	9.57E-06	31	8.16E-03	97	5.16E-01
ENSG00000149090	9.73E-06	32	1.81E-03	26	1.03E-01
ENSG00000149948	1.08E-05	33	2.13E-02	174	6.81E-01
ENSG00000164649	1.30E-05	34	3.85E-02	250	7.61E-01
ENSG00000131620	1.33E-05	35	4.68E-04	7	6.67E-01
ENSG00000162614	1.37E-05	36	1.03E-03	15	4.12E-01
ENSG00000124249	1.41E-05	37	1.18E-03	21	2.76E-01
ENSG00000112419	1.60E-05	40	6.29E-03	79	3.28E-01
